# Assessment of Hypertension Using Clinical Electrocardiogram Features: A First-Ever Review

**DOI:** 10.3389/fmed.2020.583331

**Published:** 2020-12-04

**Authors:** Kathleen Bird, Gabriel Chan, Huiqi Lu, Heloise Greeff, John Allen, Derek Abbott, Carlo Menon, Nigel H. Lovell, Newton Howard, Wee-Shian Chan, Richard Ribon Fletcher, Aymen Alian, Rabab Ward, Mohamed Elgendi

**Affiliations:** ^1^Faculty of Medicine, University of British Columbia, Vancouver, BC, Canada; ^2^Institute of Biomedical Engineering, Department of Engineering Science, University of Oxford, Oxford, United Kingdom; ^3^Research Center for Intelligent Healthcare, Coventry University, Coventry, United Kingdom; ^4^School of Electrical and Electronic Engineering, The University of Adelaide, Adelaide, SA, Australia; ^5^Center for Biomedical Engineering, The University of Adelaide, Adelaide, SA, Australia; ^6^School of Mechatronic Systems Engineering, Simon Fraser University, Burnaby, BC, Canada; ^7^Graduate School of Biomedical Engineering, UNSW Sydney, Sydney, NSW, Australia; ^8^Nuffield Department of Surgical Sciences, University of Oxford, Oxford, United Kingdom; ^9^D-Lab, Massachusetts Institute of Technology, Cambridge, MA, United States; ^10^Department of Psychiatry, University of Massachusetts Medical School, Worcester, MA, United States; ^11^Yale School of Medicine, Yale University, New Haven, CT, United States; ^12^School of Electrical and Computer Engineering, University of British Columbia, Vancouver, BC, Canada; ^13^BC Children's & Women's Hospital, Vancouver, BC, Canada

**Keywords:** ECG, digital health, cardiology, hypertension, health monitoring

## Abstract

Hypertension affects an estimated 1.4 billion people and is a major cause of morbidity and mortality worldwide. Early diagnosis and intervention can potentially decrease cardiovascular events later in life. However, blood pressure (BP) measurements take time and require training for health care professionals. The measurements are also inconvenient for patients to access, numerous daily variables affect BP values, and only a few BP readings can be collected per session. This leads to an unmet need for an accurate, 24-h continuous, and portable BP measurement system. Electrocardiograms (ECGs) have been considered as an alternative way to measure BP and may meet this need. This review summarizes the literature published from January 1, 2010, to January 1, 2020, on the use of only ECG wave morphology to monitor BP or identify hypertension. From 35 articles analyzed (9 of those with no listed comorbidities and confounders), the P wave, QTc intervals and TpTe intervals may be promising for this purpose. Unfortunately, with the limited number of articles and the variety of participant populations, we are unable to make conclusions about the effectiveness of ECG-only BP monitoring. We provide 13 recommendations for future ECG-only BP monitoring studies and highlight the limited findings in pregnant and pediatric populations. With the advent of convenient and portable ECG signal recording in smart devices and wearables such as watches, understanding how to apply ECG-only findings to identify hypertension early is crucial to improving health outcomes worldwide.

## Introduction

Hypertension is a major cause of morbidity and mortality (1) and affects an estimated 1.4 billion people worldwide (2). Hypertension is defined as blood pressure (BP) that is elevated above 130 mmHg systolic or above 80 mmHg in stage 1 and above 140 mmHg or above 90 mmHg in stage 2 by the American College of Cardiology and American Heart Association (3). Long term consequences of hypertension can include ischemic heart disease, stroke, and end stage renal disease (1). Cardiac structural changes from prolonged hypertension can lead to arrhythmia and sudden cardiac death (4). Managing BP early, even at prehypertensive levels, may decrease the incidence of cardiovascular events later in life (5). Recognizing and intervening in acute BP emergencies can also decrease mortality (6). Hypertensive conditions in pregnancy, such as pre-existing hypertension, gestational hypertension, preeclampsia, and eclampsia, can lead to perinatal and maternal morbidity and mortality (7). All of these findings highlight the importance of having an effective BP surveillance system.

Prehypertension and primary hypertension can be difficult to identify, as their diagnosis relies on BP readings (3). Conventionally, BP is measured using an auscultatory sphygmomanometer or automated oscillometric devices in health care settings or with oscillometric devices at home (3). Obtaining accurate BP measurements are challenging as it is necessary to coordinate multiple factors with the patient such as ensuring the patient has emptied their bladder, has relaxed for at least 5 min and has avoided smoking, exercise, and caffeine 30 min prior (3). Furthermore, the BP cuff, the BP device type, the measurer's technique, and the frequency of BP measurements also need to be considered (3). These measurements take time and training for health care providers and are inconvenient for patients who need to visit a doctor's office or learn to manage their own BP monitoring at home. In addition, errors in BP readings are made by both physicians and patients (8, 9). Even if health care providers and patients meet all the measurement guidelines, the measurement methods only capture a few instantaneous readings of BP for analysis. Further, numerous daily variables can affect BP from moment to moment (3, 8, 10). This is concerning, as even a 5 mmHg difference to the measurement can change a patient's BP category and management plan (10). Thus, there is an unmet need for an accurate, 24-h continuous and portable BP measurement system.

There has been ongoing work to develop alternative, more convenient ways of measuring BP using photoplethysmography (PPG) and electrocardiograms (ECGs or also known as EKGs) (14–18). These methods range from using a single biomedical signal to assess BP (17), to using multiple body sites with PPG input (15), to using PPG along with another type of input such as ECGs (18). However, measuring BP using only ECG signals has not been reviewed. The aim of our review is to identify if there are specific ECG features that could be used alone to measure BP or identify hypertension.

Note that the ECG waveform can provide valuable information about the heart status. The waveform features can be analyzed to determine, for example, heart rate variability and left ventricular hypertrophy. It can also be used to design machine-learning algorithms for other BP studies. Links between hypertension and such biomedical signals may show promise for monitoring BP.

It is hypothesized that elevated heart rates represent persistent sympathetic activation, which leads to increases in BP and cardiovascular complications (19). Heart rate variability (HRV) can be estimated from ECG signals and can reveal whether there is dysregulation of the autonomic system leading to a decrease in the ability to return to lower heart rates (19, 20). HRV may show some promise in monitoring hypertension and poor pregnancy outcomes as HRV has been found to decrease as BP increases (20–24). However, HRV can be measured in different ways and requires its own review outside of this paper.

Left ventricular hypertrophy (LVH) is thought to be an adaptive response to hypertension and is found in a small portion of hypertensive patients at 5–18% using ECG signals (23, 25). It has been used to predict dangerous cardiac arrhythmia and other cardiovascular risks in hypertensive patients (23, 25). Note that LVH is generally a long term complication for elevated BP over a long period of time (4), which may make it less promising finding to identify new onset hypertension.

Some early work has looked at using machine-learning to identify ECG signal features that can potentially categorize individuals as high-risk hypertension (SBP ≥ 130, DBP ≥ 80) or low-risk hypertension (SBP < 130, DBP < 80) (26). Note that authors did not mention the logical operation between the SBP and DBP, in other words it was not clear if it is an “OR” or “AND” between them. ECG signal components may be mapped (or interpreted) into a quantitative number based on machine-learned algorithms and considered for the identification of and monitoring of chronic conditions (i.e., pulmonary artery hypertension, hypertension, coronary artery disease, etc.) (27). Additionally, ECG signal components may be categorized into hypertension (SBP ≥ 140 OR DBP ≥ 90), prehypertension (SBP = 120–139 OR DBP = 80–89) or normal BP (SBP < 120 AND DBP < 80) and then machine learning with this categorization may predict specific BP values for individuals (28). However, there are few studies in this field to draw conclusions from at this time.

This review focuses on analyzing ECG wave morphology without HRV, LVH or machine learning. ECG wave morphology has been a promising area of investigation related to hypertensive individuals. In a review of ECG parameters in the context of hypertension and arrhythmia risk, P wave duration and QT dispersion were noted to have decreased after BP was controlled with medication (23). In addition, T wave amplitudes have been noted to differ between hypertensive patients and normotensive individuals (23) However, there have been no detailed reviews specifically looking at ECG wave morphology differences between hypertensive and normotensive individuals. Interestingly, ECG wave morphology changes can happen over a short period of time, such as from before to after treatment of a hypertensive crisis (29). Therefore, ECGs may provide 24-h continuous opportunity for monitoring of hypertension and for alerting patients and their health care providers to acute conditions, such as hypertensive emergencies or preeclampsia.

In this current review, we conducted a search of literature published from the last decade on the use of only ECG wave morphology to either measure BP or identify hypertension. The aim of this review is to summarize the current understanding of monitoring BP, identifying hypertension using only ECG wave morphology and provide an effective BP surveillance system. We set our ultimate review question to be: can we use only ECG wave morphology to measure BP or identify hypertension in humans?

## Methods

### Search Strategy

On March 27, 2020, we searched PubMed for articles with keywords related to ECG wave morphology and BP measurement using “AND.” We also included “EKG” as a keyword as it is equivalent to ECG. We filtered our searches to focus on the title/abstract. We searched the last 10 years (January 1, 2010, to January 1, 2020) to determine the current understanding of the topic. These initial searches resulted in 2,951 articles.

We excluded any articles that were reviews, case studies, or commentaries. We also filtered out any articles that were not in English. As we focus on only ECGs, we used “NOT” to remove articles with words related to photoplethysmography technology, which we are aware has been used in combination with ECGs to measure BP. To narrow the search to articles looking at ECG wave morphology as a way to measure BP, we identified ECG parameter related words in a quick exploratory literature search and combined these words with “AND.” The exact search terms are described in Appendix 1, and the final search entry is described in Appendix 2.

### Study Selection and Study Data Extraction

**Figure 1:**
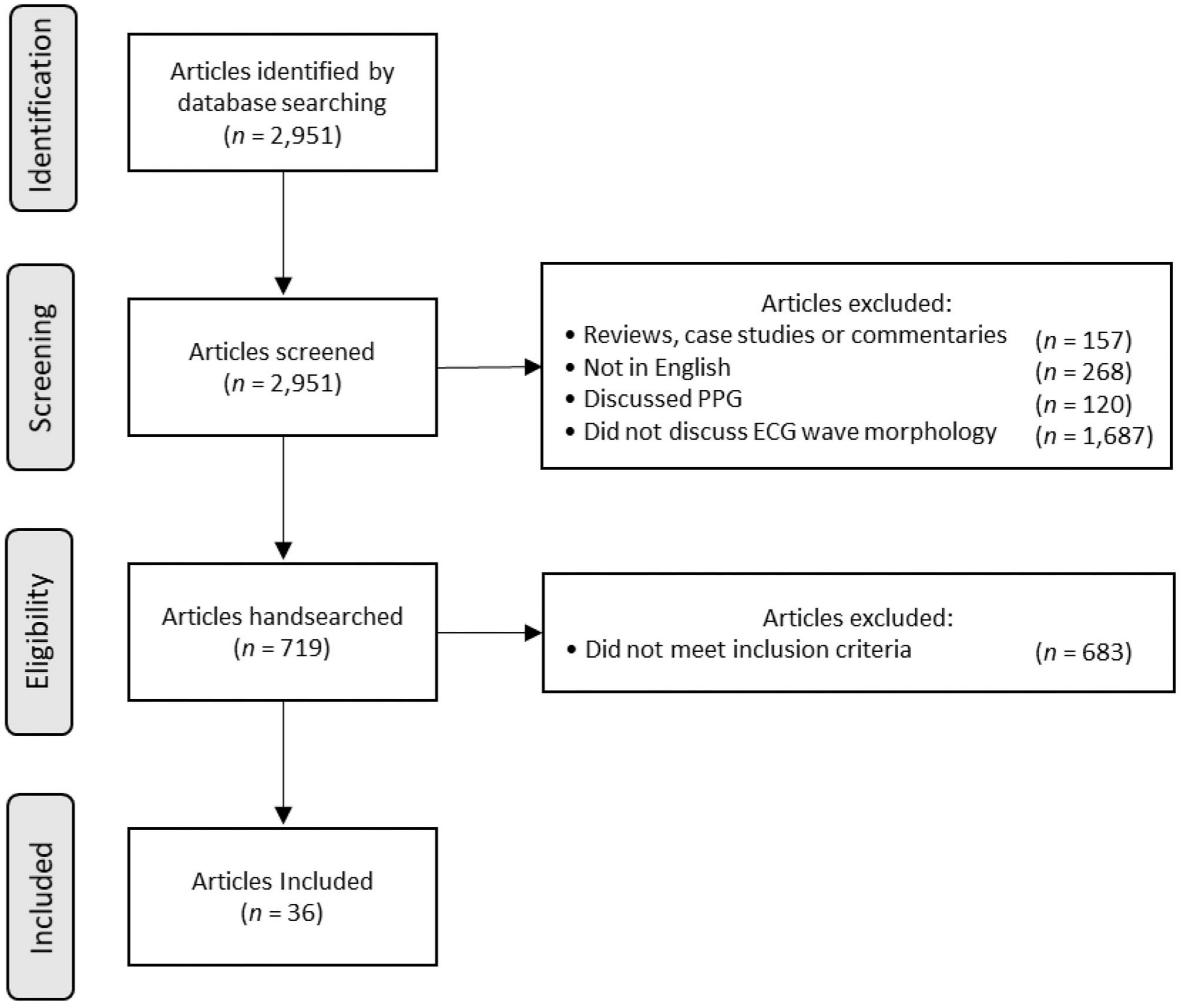
Flowchart of the article search, screening, and selection process.

From January 1, 2010, to January 1, 2020, we identified 719 articles from our search. We then hand-selected articles if their title or abstract indicated that their primary aim was to use only ECG wave morphology to measure BP or identify hypertension. If ECGs were combined with another method, we did not select the article. The flowchart of the inclusion and exclusion is shown in Figure 1.

Several articles mentioned prediction of a future diagnosis of hypertension or assessment of participants with hypertension to predict if they are at low or high risk for complications from hypertension, such as myocardial infarction, stroke, or atrial fibrillation. We did not select these articles as we are not looking to predict hypertension or strategize about the risk of those with hypertension. Instead, we focus on measuring BP or diagnosing hypertension in the present. We also did not select articles which used non-clinical ECG features such as ECG segments found through machine learning. We are interested in ECG features that can be identified in the clinical setting.

We selected articles in which participants were recruited from the general population or based on their blood pressure. We avoided articles that selected their participant populations based on other medical conditions, such as diabetes mellitus, metabolic syndrome or obesity (defined as Body Mass Index (BMI) over 30 kg/m^2^) (30). This was done to avoid confounding medical conditions that may alter ECG morphology. We also used articles with human participants of any age to gain a comprehensive understanding of the age groups that have been studied.

In total, 36 articles that fit our inclusion criteria and are listed in Appendix 3. We fully read 36 articles and extracted data regarding the participants (e.g., sample size, sex, and age), BP measurement systems, ECG parameter estimation methods, and the articles' conclusions about the significance of ECG wave parameters. We collected all participant data up to one decimal place and all *p*-values up to four decimal places if they were available.

Based on our review, there were two main ways to measure BP: cuffed with discrete measurements and cuffless with continuous measurements. We listed if each study used a cuffed or cuffless method in the table in Appendix 3. For the cuffed measurements, they were intermittent and non-invasive using a cuff to collect a discrete BP measurement at set time points. All the articles used cuffed measurements. One article used a cuffed method and confirmed if individuals were hypertensive or normotensive with the cuffless method. The cuffless method used a continuous and invasive method by cannulating the radial artery for inter-arterial BP measurements (31).

For the BP categorization of participants, some articles had different definitions of hypertension, prehypertension and normotension and some articles grouped participants just by different increments of BP measurements. We listed the definitions of BP categorization used in each article in Appendix 3. One study had data for both SBP and DBP (13). We used the SBP *p*-values from this article.

For ECG parameter estimation methods, we categorized them as either “manual” (when the study specifically said they used “manual” methods, used physical instruments such as paper, calipers, rulers or when multiple individuals were involved in estimating ECG parameters), “Computed” (when computer software automatically estimated ECG parameters), “mixed” (when both manual and digital methods were involved in estimating ECG parameters; not including when individuals simply checked if the ECG recording was of satisfactory quality) or “NR” (when the details were not reported or if it was unclear if manual or computed methods were used).

There were two articles with the same first author that had identical participant numbers, and ECG results. We removed one of these articles to avoid adding duplicate data into our review. This removed article is listed after the included article in Appendix 3. All 36 articles were reviewed and verified and 35 articles were used for analysis.

### Data Analysis

Using the extracted data, we grouped the 35 articles' findings into tables based on ECG wave parameter type. We then analyzed the articles based on their sample size, the participants' comorbidities and confounders, the number of participants in each BP class, the definitions the articles used to categorize BPs, the gold standard the articles used to measure BPs, the ECG leads used, the sampling frequency, how ECG parameters were estimated and the participants' position during BP and ECG signal collection (i.e., sitting, supine, etc.). If the participant data was split up into different subgroups, such as by BP categorization, we used the following Cochrane formula to combine mean and standard deviation across two groups to derive an integrated value (32):

$$\begin{array}{l} {\bar{x}}_{p}=\frac{N_{1}M_{1}+N_{2}M_{2}}{N_{1}+N_{2}},\\ {\tilde{x}}_{p}=\frac{\sqrt{\left(N_{1}-1\right)SD_{1}^{2}+\left(N_{2}-1\right)SD_{2}^{2}+\frac{N_{1}N_{2}}{N_{1}+N_{2}}\left(M_{1}^{2}+M_{2}^{2}-2M_{1}M_{2}\right)}}{N_{1}+N_{2}-1}, \end{array}$$

where, ${\bar{x}}_{p}$ is pooled mean, ${\tilde{x}}_{p}$is pooled standard deviation, *N*_1is_ the number of participants in the first group, *N*_2_ is the number of participants in the second group that is being combined with the first group, *M*_1_ is the mean value of *x* in the first group, *M*_2_ is the mean value of *x* in the second group, *SD*_1_ is the standard deviation for the first group and *SD*_2_ is the standard deviation for the second group. When there were more than two groups to combine, the first and second group would be combined. Then the resulting value would be considered as a “new” first group. The third group would then be considered as a “new” second group for the application of the above formulas.

There was one article by Anigbogu et al. (33) which seemed unclear if a figure was missing indicators of significant results for certain ECG parameters. We added double asterisk to the ECG parameters in question in Appendix 3 and have treated the results as not significant.

With each ECG wave parameter, we first analyzed the overall theme of the article, such as if there was a consensus on whether the features of the parameter had a significant ability to predict SBP or DBP or to identify individuals with elevated BP. We considered an article to have a significant result if the *p*-value was less than 0.05. For cases when there were two or less articles regarding a certain feature, then this feature was not analyzed due to the scarcity of the number of studies. For example, the P wave duration was only investigated by two articles that yielded confounding results; thus, resulting in a lack of identifiable patterns. Contrarily, the P wave maximum was investigated by 10 separate studies which granted the opportunity to analyze the findings and determine whether there was a consistent pattern demonstrating ECG changes in hypertensive patients.

Second, we grouped articles based on whether the participants had comorbidities such as diabetes mellitus, metabolic syndrome, and an average BMI in the obese range as described earlier. We also considered confounders, such as whether participants smoked or had significant differences in alcohol consumption, age, BMI or sex, as these variables have been noted to alter ECGs (34–41). Ultimately, we looked at whether there was a difference in the findings when there were known comorbidities and confounding factors present and when the participants were healthy with no comorbidities or confounding factors. When we only analyzed articles without comorbidities or confounders, there were only eight articles present, so we focused on features if there were two or more articles finding significance to be able to provide results with this point of view.

Third, we investigated the groupings of articles with pregnant participants and with pediatric participants. This was done to determine if there were any promising ECG parameters in these populations.

Fourth, we investigated groupings of ECG parameters (i.e., atrial and ventricular parameters) to determine if there was a consensus about whether elevated BPs are more commonly present with atrial or ventricular changes. We focused on using data from articles without comorbidities or confounders.

Fifthly, we listed how many leads were used in each study. If a specific lead was focused on for an ECG parameter, we also listed this in our tables. This is to investigate if significant results can be obtained from using fewer leads than a standard 12-lead ECG.

Finally, we categorized each article by if they used manual, digital, mixed or did not report how their ECG parameters were estimated. We investigated if there was a trend in how ECG parameters were being estimated.

### Hypothesis

We hypothesize that our review will find ECG parameters that are consistently differentiated between normotensive and hypertensive individuals.

## Results

**Figure 2:**
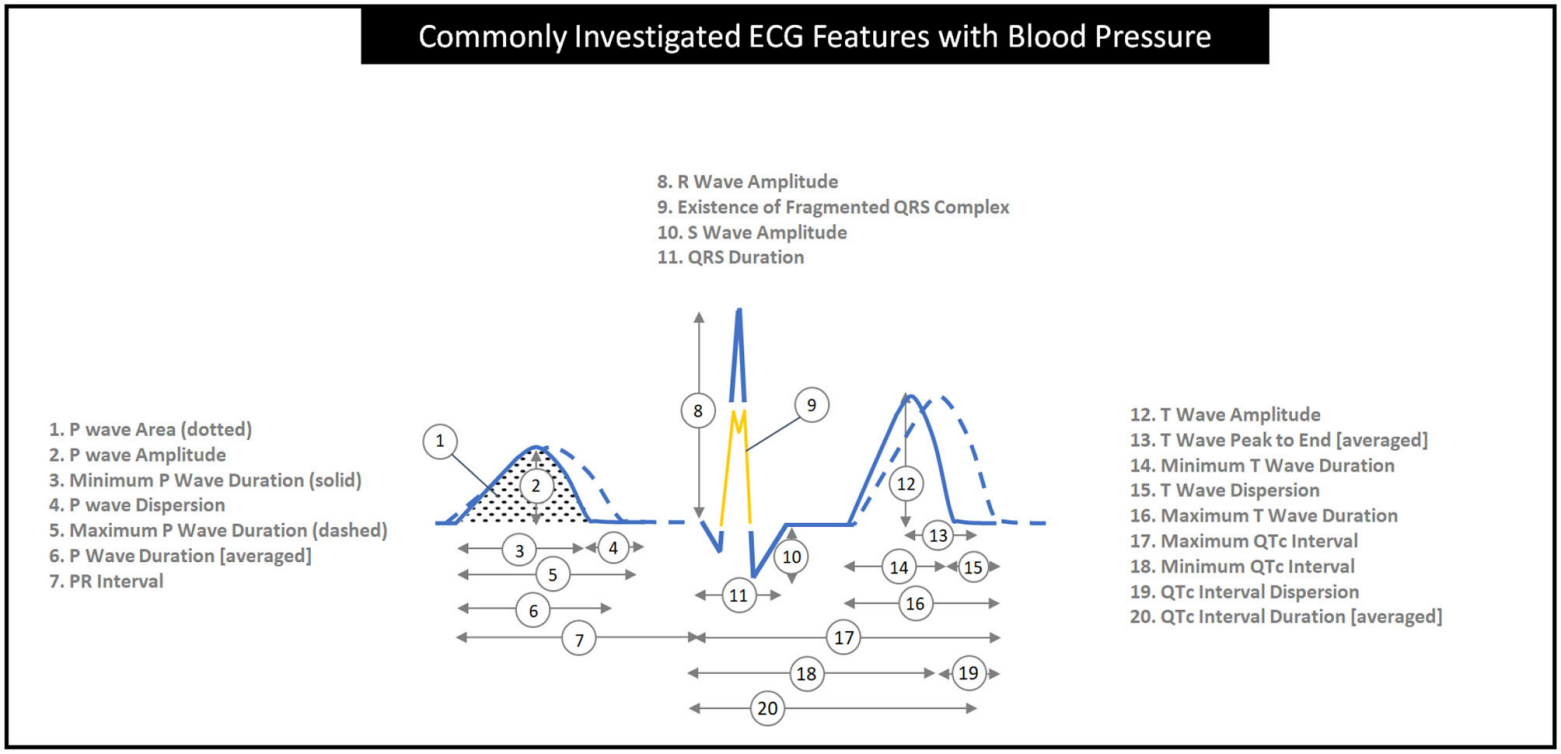
The graphical definition of common ECG wave features seen in this review of 35 articles. QT intervals in numbers 8 to 11 are corrected for heart rate into QTc intervals by applying a formula such as Bazett's (QTc = QT/$\sqrt{RR\;interval}$) (11) or Hodge's formula [QTc = QT + 1.75 (heart rate – 60)] (12). Note that the two ECG signal segments (solid and dashed) are collected from the same subject.

Between January 1, 2010, and January 1, 2020, we selected 36 articles and analyzed 35 articles which investigated the use of only ECG wave morphology parameters (not HRV indices, LVH measurements or machine learning) to measure BP or identify hypertension. Two articles had identical participant numbers and ECG results, so one of these articles was removed. Appendix 3 presents the full list of the 35 articles analyzed in this paper and the one removed article. Appendix 4 groups the articles based on their ECG wave parameter findings. Based on the analyzed articles, a summary of commonly used ECG features for assessing hypertension is shown in Figure 2. The definition of each feature is described in Table 1.

**Table 1:** Summary of ECG wave morphology parameter definitions.

**Feature**	**Definitions**
P wave maximum	Longest P wave across all leads (in milliseconds) (13)
P wave minimum	Shortest P wave across all leads (in milliseconds) (42)
P wave duration	Average P wave duration in each all lead (in milliseconds) (42)
P wave prolongation	P wave longer than 120 ms (43)
P wave dispersion	Difference between P wave maximum and P wave minimum (in milliseconds) (13)
P wave area	Total area under and above the isoelectric line from the beginning to the end of the P wave (13) OR The product of P-wave amplitude per half of lead II duration (42)
P wave amplitude	Amplitude of P wave (in millivolts) (42)
P wave terminal force	“Product of the duration (in seconds) and amplitude (in millimeters) of the negative terminal deflection of the P-wave” (44)
PR interval	Length between the beginning of the P wave to the beginning of the QRS complex (13)
QT maximum, minimum, dispersion and duration	Similar to P wave above. QT interval is the beginning of the Q wave until the end of the T wave (45). QT dispersion is the difference between QT maximum and QT minimum (in milliseconds) (45)
Abnormal QT dispersion (QTd)	QTd greater than the total of the mean QTd plus two standard deviations of the normotensive group (46)
QTc maximum, minimum, dispersion, duration	Similar to P wave above. QTc = QT interval corrected for heart rate using Bazett's formula: QT/$\sqrt{RR\;interval}$ (11) Only Kirbas et al. (12) uses Hodges formula instead to correct for heart rate: [QTc = QT + 1.75 (heart rate – 60)]
QTc prolongation	QTc is longer than the mean QTc plus two standard deviations of the normotensive group for each sex (46) OR Longer than 440 ms for Akintunde et al. (45) OR Longer than 430 ms in males and longer than 450 ms in females for Solanki et al. (47) OR Longer than 450 ms in males and longer than 460 ms in females for Sun et al. (48)
QTcF duration (ms)	Uses the Fridericia formula which takes the QT interval and divides it by the cube root of the RR interval to correct for heart rate (13)
QTfr maximum (ms)	Uses Fridericia formula which takes the QT maximum interval and divides it by the cube root of the RR interval to correct for heart rate (49)
QTc peak	Time from start of the QRS complex until the point of a positive T wave peak (50) Corrected for heart rate using Bazett's formula (50)
Fragmented QRS	QRS complexes that have notches in the R or S wave or an additional R wave or more in two contiguous leads (51)
QRS duration	Average of the first deflection from the isoelectric line after the P wave until the J-point (13)
QRS axis	Not defined (45)
R wave amplitude	Amplitude of R wave (in millivolts) (52)
R wave axis	“Net vector of the R wave axis based on the extremity leads” (in degrees) (13)
Poor R wave progression	“R wave in the precordial lead V3 ≤ 3 mm and R in lead V2 ≤ R in lead V3” (53)
S wave amplitude	Amplitude of S wave (in millivolts) (52)
SV1/V2 + RV5/6	Amplitude of S wave in lead V1 or V2 added to the amplitude of R wave in lead V5 or V6 (in millivolts) (45)
T wave maximum, minimum, dispersion, amplitude	Similar to P wave above (13)
T wave alternans	Changes in the shape, amplitude or timing of T waves or ST segment (54)
T wave axis	T wave axis was divided into abnormal and not abnormal T wave axis. An abnormal T wave axis was – 180° ≤ T wave axis < – 15° or 105° < T wave axis ≤ 180° (55)
TpTe	Length between peak of T wave to the end of the T wave (13)
TpTe(c)	TpTe corrected for heart rate using Bazett's formula (50)
TpTe/QT TpTe/QTcTpTe(c)/QTc	TpTe or TpTe(c) divided by QT or QTc for a ratio as a possible “marker of the ventricular heterogeneity of repolarization” (56)
Ventricular Activation Time (VAT)	“First deflection from the isoelectric line following the P wave until the peak of the R wave” (in milliseconds) (13)
J point T peak duration (corrected for heart rate)	“Duration of QRS complex offset to peak of the T wave/RR interval. RR interval is the interval between the onset of one QRS complex to the onset of the next QRS complex, measured in seconds, derived from the heart rate (HR) as 60/HR” (13)
Ischemic ECG abnormalities	“Presence of ST and T wave abnormalities suggestive of ischemia, ischemic T wave changes, abnormal Q/QS waves, and the presence of left bundle branch block” (57)

*C, corrected for heart rate*.

### Comparing ECG Wave Morphology

There were 14 articles that investigated P wave features, as seen in Table A. Note that P wave maximum and dispersion were the most commonly investigated features, with ten articles each. In seven of the 10 articles, P wave dispersion was found to be significantly greater with elevated BP. The three non-significant articles had lower average BMIs (22.7, 26.1, not reported vs. 23.9 to 29.7 in significant articles), populations who had comorbidities excluded, participants only recently diagnosed with hypertension, or did not have hypertensive participants (13, 42, 56). In five of the 10 articles, the P wave maximum was significantly higher with elevated BP. The five articles with the differences between HT participants and NT participants noted differences ranging from 2.8 to 23.3 ms (44, 58–61). The non-significant articles had populations that had younger average ages (22.7 to 51.6 vs. 44.7 to 53.6 years old), healthier with none or less comorbidities listed, and all of them were recently diagnosed with hypertension or did not have hypertensive participants (13, 42, 56, 62, 63). The P wave minimum was the least significant, with only one article of seven reporting a significant finding. P wave duration, prolongation, area, amplitude, and terminal force were all investigated by two or fewer articles. It can be seen that PR intervals were significantly longer with increased BP in two of six articles, as shown in Table B. The four studies that were non-significant had populations which were generally younger 22.7, 42.8, 56.0, and not reported compared to 54.2 and 55.6 years old, had lower BMIs 22.7 to 26.5 compare to 28.2, had healthier participants with comorbidities removed and three of the four had participants with newly diagnosed hypertension or no participants with hypertension (13, 33, 42, 45).

QT and QTc intervals were included in 15 articles, as shown in Table C. In three of four articles, QT dispersion was found to be significantly larger with higher BP. An example of the most substantial difference was an article with a mean QT dispersion of 65.6 ms for HT participants and 38.7 ms for NT participants (46). The article that did not find significance had a younger population average age of 28.2 years old vs. 42.8, 56.0, and 57.4 years old (56). QT duration was not significant in three out of three studies. Two or fewer articles looked at other QT features, such as QT maximum, QT minimum, and abnormal QT dispersion. The most articles analyzed QTc duration, and five of nine articles found it was significantly longer with increased BP. With the four articles which did not find significance, there was slightly lower BMI (26.1 to 26.5 vs. 27.4 to 30 for significant articles), but otherwise no clear differences in age, gender, number of participants with elevated BPs, number of comorbidities, BP measuring technique or ECG analysis technique (31, 42, 45, 50). All three articles on QTc dispersion found that it was significantly greater with higher BP. QTc prolongation was also significant in all four articles. Two of the four articles on QTc maximum showed that it was significantly longer with higher BP. The two articles which did not find significance either had no participants with hypertension (11), or the participants were young at 28.2 years old, healthy without comorbidities, and recently diagnosis in pregnancy (56). Kirbas et al. did find a significant difference with a young healthy pregnant population as well, but they only found significance when they were looking at severe preeclampsia which has a BP equal or over 160/110 mmHg or systemic symptoms (12). They did not find significance with mild preeclampsia (12). There were no significant findings for QTc minimum in three articles. QTc peak, QTcF duration and QTfr Maximum over 50 ms were all mentioned in two or fewer articles.

Twelve articles studied the QRS complex, as shown in Table D. Fragmented QRS was significantly more present with elevated BP in three of three articles. The QRS duration was significant in two of four articles. The two articles without significance had older population averages in the 40 s compared to 22.7 years old and not reported and higher BMI averages at 26.1 and 29.4 compared to 22.7 and not reported (42, 64). The R wave amplitude was significantly greater in three of three articles. There were two or less articles for QRS axis, Q/QS wave abnormality, R wave axis, poor R wave progression, S wave amplitude and adding S wave to R wave amplitudes.

Six articles looked at T wave features, as shown in Table E. No more than two articles investigated T wave maximum, minimum, dispersion, amplitude, electrical alternans, T wave axis and T wave inversion. T wave amplitude was the most notable T wave feature, and two of two articles found that it was significant for higher BP.

Seven articles investigated the time from T wave peak to the end of the T wave (TpTe), as seen in Table F. Note that TpTe was significantly longer, as BP increased in four of five articles. The one article that did not find significance had a young average age of 22.7 years old, population that was recruited to be free from any comorbidities, the lowest BMI average in the five articles of 22.7, the lowest percentage of females at 26.3% and only 17.4% participants had prehypertension with no hypertensive participants in the study (13). Two or fewer articles mentioned TpTe maximum or variations in TpTe which corrected for heart rate or divided it by QT or QTc.

Other ECG morphologies are mentioned in Table G. Queen et al. (57) grouped a number of ECG morphologies into an “ischemic ECG abnormalities” group. Two articles looked at ventricular activation time (VAT). One article looked at J point T peak duration corrected for heart rate. All of these articles found significant results except for one VAT article. Anigbogu et al. did not find any significant difference in ST duration between hypertensive and normotensive participants (33). Sriratanaviriyakul et al. investigated multiple features such as ST depression, atrioventricular conduction defects and ventricular conduction defects, however only found a small significant difference in right bundle branch blocks presence in hypertensive participants compared to normotensive participants (65).

### Grouped by Presence or Absence of Comorbidities and Confounding Factors

Of the 35 articles used in this paper, 26 articles identified comorbidities or confounders, such as diabetes mellitus, metabolic syndrome, alcohol consumption, smoking, and obesity with a mean BMI over 30 kg/m^2^ within their populations. We compared the ECG wave parameters between articles with and without comorbidities and confounders.

**Figure 3:**
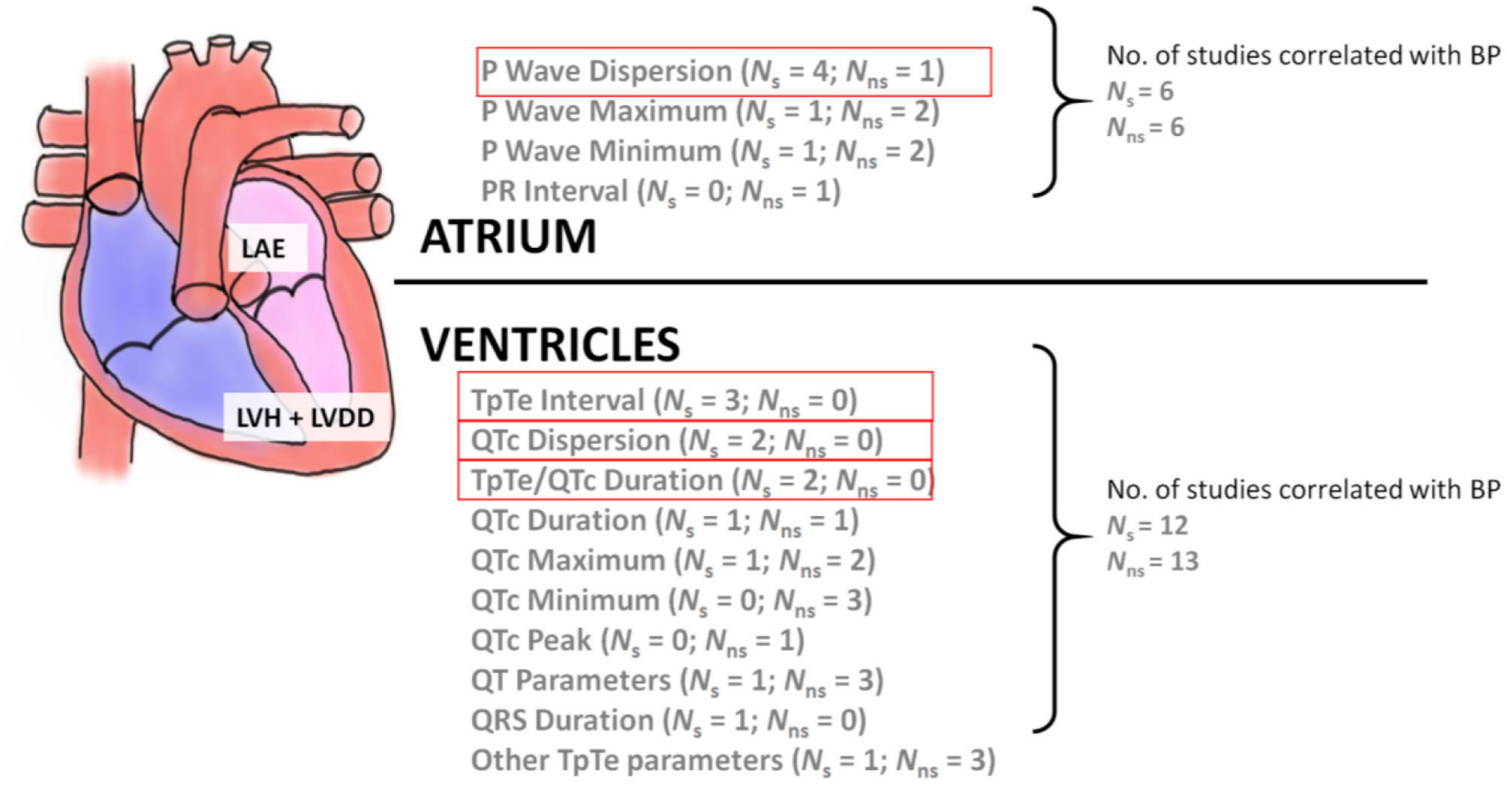
The relationship between atrial and ventricular ECG wave features with BP. LAE, left atrial enlargement; LVH, left ventricular hypertrophy; LVDD, left ventricular diastolic dysfunction. Note that *N*_s_ stands for the number of studies of statistical significance (*p* < 0.05) while *N*_ns_ stands for the number of studies of non-statistical significance. The four features highlighted in red show features that have two or more studies with statistically significant results.

In the five articles without comorbidities or confounders for P wave features (56, 58, 62, 66, 67), only P wave dispersion still looked promising with four of five articles with demonstrating significant findings (58, 62, 66, 67). QTc dispersion was significant in two of two articles without comorbidities or confounders (11, 12). The T wave peak to the end of the T wave interval (TpTe) was significantly longer in three of three articles without comorbidities or confounders (11, 12, 56). The most profound difference in TpTe was 93.1 ms for PHT participants and 67.9 ms for NT participants (11). TpTe/QTc ratio was also significantly different in two of two articles (12, 56). A summary of grouping ECG features associated with hypertension in terms of atrium and ventricles is shown in Figure 3. The other ECG wave parameters were not considered as they did not have more than one significant result in the group of articles without comorbidities or confounders.

### Special Populations

Articles with pregnant participants [three articles (12, 56, 62)] and pediatric participants [two articles (66, 67)] are considered as special populations in this review. No ECG parameters were investigated by all three articles with pregnant populations (12, 56, 62). Gazi et al. (56) and Kirbas et al. (62) both looked at P wave parameters. Kirbas et al. (62) reported significant P wave dispersion and P wave minimum. Gazi et al. (56) did not find any significance in its P wave maximum, minimum, or dispersion. A different article by Kirbas et al. (12), reported a significant increase in QTc dispersion, maximum, and duration when normotensive pregnant women were compared to pregnant women with severe preeclampsia. In comparing mild preeclampsia with normotensive pregnant women, only QTc dispersion was significantly increased. Gazi et al. (56) found significance for QT maximum, but did not find any significance for QTc maximum. Both Kirbas et al. (12) and Gazi et al. (56) found that TpTe and TpTe/QT ratio were significantly increased in pregnant women with pregnancy-induced hypertension when compared to normotensive pregnant women.

The two pediatric articles measured BP after the participant rested for 10 min and an appropriately sized BP cuff was used (66, 67). These articles classified normotensive participants into the 90th percentile for their sex, age and height group, prehypertensive as between the 90th to 95th percentile, and hypertensive as above the 95th percentile. Both of the pediatric population articles only studied P wave dispersion and found that it was significantly increased as BP increases (66, 67). One of these two articles noted that 16.8% of its participants were obese (66), and the other article did not mention the BMI measurements of the population (67).

### Atrial vs. Ventricular Parameters

Atrial parameters included P wave parameters and PR interval. Looking at both of these parameters in articles without comorbidities or confounders, there were six significant results and six not significant results. It is unclear if atrial parameters overall change with elevated BP compared to normal BP. Ventricular parameters included QT/QTc, QRS complex, R wave, S wave, T wave, and TpTe. Only QT/QTc and TpTe parameters were available in articles without comorbidities or confounders. There were 12 significant results and 13 not significant results in these ventricular parameters. As with atrial parameters, it is also unclear whether ventricular parameters are significantly changed by increased in BP. A summary of these atrial and ventricular parameters without comorbidities or confounders can be found in Figure 3.

### ECG Parameter Estimation

ECG parameter estimation methods are listed in the last column in Appendix 3. Twenty articles used manual methods, six mixed manual and automatic computation with software methods, six used only computed methods and three articles did not specify how ECG parameters were estimated. Five articles described their manual methods such as using calipers and a magnifying glass to identify ECG deflections (12, 63). Fourteen articles stated they used a “manual” method without further details about instruments used (56, 68). One article scanned the 12-lead ECG recording into a computer at 600 dpi and two investigators used a computer program to manually measure the ECG parameters on the computer (42). Manual methods were either described as being done by cardiologists (seven articles), investigators (four articles), a general practitioner and a cardiologist (one article), a physician (one article), experts (one article), observers (three articles) or not specified (three articles). Manual methods had either two individuals (11 articles), one individual (five articles), three individuals (one article) or an unspecified number of individuals estimating ECG parameters (three articles).

The mixed methods noted cardiologists (two articles), reviewers (two articles), or did not specify (two articles). The mixed methods either noted only one person manually involved (three articles) or did not specify how many people were involved (three articles). In the manual and mixed method categories, 12 articles noted that the individuals estimating ECG parameters were blinded to the participants' clinical statuses and 14 did not report if the individuals were blinded or not. The computed methods used different automated software programs for estimating ECG parameters except for a software that was used by two articles (13, 61) and both Sun et al. articles used the same software between them (43, 48).

## Discussion

In total, 35 articles published from January 1, 2010, to January 1, 2020 were analyzed in this review for using only ECG wave morphology to identify participants with elevated blood pressure. Regarding the ECG wave parameters, P wave parameters were the most commonly investigated feature in our review. These parameters include P wave maximum, minimum, duration, prolongation, dispersion, area, terminal force and amplitude as well as PR interval. Previous studies have shown that P wave changes predict left atrial enlargement (LAE), which can occur in various conditions (69). In cases of hypertension, LAE occurs as a compensatory reaction to decreased left ventricular compliance as a result of chronically increased left ventricular pressure, left ventricular hypertrophy, and diastolic dysfunction (70). However, some literature questions the utility of P wave morphological analysis for identifying LAE (71, 72).

In our review, P wave dispersion appeared the most promising for monitoring BP according to the number of studies in which it was significantly present in cases of higher BP. The literature supports monitoring P waves; for example, Aksoy et al. (29) treated 30 participants for hypertensive crises and noted that P wave parameters could change acutely upon treatment, finding significant decreases in P wave maximum and dispersion between the time of presentation and the period after treatment. In addition, Çimen et al. noted that P wave dispersion and maximum were significantly present before cardiac structural changes (73). However, in our review, when articles with comorbidities and confounders were removed, many significant P wave parameters were also removed except for P wave dispersion. The remaining P wave dispersion articles can be seen in Table 2. It is notable that most of the studies without comorbidities or confounders were in populations with children or pregnant women. This wide variety of population characteristics in only a few studies prevents us from being able to draw a conclusion about P wave parameters at this time.

**Table 2:** Summary of the four ECG wave parameters with the greatest number of articles without comorbidities or confounders that found significant changes with elevated blood pressures.

**ECG wave parameter**	**Number of articles significant vs. not significant**	**Articles**	**Number of participants**	**Pregnant population**	**Gold standard BP method**	**Results from articles for NT vs. HT/PIHT**		***p*-value**
P wave Dispersion	4 articles significant 1 article NS	Gazi et al. (56) Kirbas et al. (62)	41 88	Yes Yes	Cuff-based Cuff-based	NT: 33.6 ± 8.9 ms NT: 42.1 ± 17.0 ms	PIHT: 32.3 ± 10.1 ms PIHT: 60.3 ± 17.7 ms^*^	0.516 <0.05
		Chávez et al. (66)	656	No	Cuff-based	NT: 30.1 ± 10.5 ms	HT: 39.1 ± 11.5 ms	<0.001
		Chávez et al. (67)	515	No	Cuff-based	NT: 31.9 ± 9.3 ms	HT: 39.7 ± 11.6 ms	<0.001
		Yildirim et al. (58)	48	No	Cuff-based	NT: 35.5 ms (STD NR)	HT: 43.5 ms (STD NR)	<0.05
QTc Dispersion	2 articles significant 0 article NS	Kirbas et al. (12) Tanindi et al. (11)	96 84	Yes No	Cuff-based Cuff-based	NT: 46.8 ± 10.7 ms NT: 27.2 ± 5.2 ms	PIHT: 74.5 ± 11.4 ms^*^ PHT: 36.1 ± 6.8 ms	<0.001 <0.001
TpTe Interval	3 articles significant 0 article NS	Gazi et al. (56) Kirbas et al. (12)	41 96	Yes Yes	Cuff-based Cuff-based	NT: 75.8 ± 8.4 ms NT: 74.1 ± 9.4 ms	PIHT: 83.5 ± 7.8 ms PIHT: 97.5 ± 5.9 ms^*^	0.007 <0.001
		Tanindi et al. (11)	84	No	Cuff-based	NT: 67.9 ± 8.1 ms	PHT: 93.1 ± 7.6 ms	<0.001
TpTe/QTc Ratio	2 articles significant 0 article NS	Gazi et al. (56) Kirbas et al. (12)	41 96	Yes Yes	Cuff-based Cuff-based	NT: 0.18 ± 0.02 NT: 0.18 ± 0.01	PIHT: 0.19 ± 0.02 PIHT: 0.23 ± 0.01	0.037 <0.001

*NS, not significant; NT, normotensive; PHT, prehypertensive; PIHT, pregnancy-induced hypertension; HT, hypertensive; ms, milliseconds; STD, standard deviation; NR, not reported*.

* *Severe preeclampsia group. ${\bar{x}}_{p}=$ calculated pooled mean of variable x, where x can be BMI or age*.

There is a large body of research that supports P wave changes in the presence of various comorbidities and confounders, such as sex, age, obesity, smoking habit, and diabetes mellitus (36, 43, 74–79). With this in consideration, the participants in Aksoy et al.'s study had an average BMI of almost 30 kg/m^2^ (29), and 42% of participants in Çimen et al.'s study smoked (73). These confounders may have impacted the P wave results. There are also racial differences; for example, South Asians have been reported to have reduced P wave dispersion (80). Furthermore, it is reported that P wave changes only occur after prolonged exposure to hypertension and the appearance of complications, such as atrial fibrillation (81, 82). Overall, P waves may not be dependable for specifically monitoring elevations in BP, but further studies without comorbidities and confounders are needed.

There were both two significant and four not significant results for PR intervals in the six articles that investigated PR intervals. There was one not significant article involving PR intervals when articles with comorbidities and confounders were removed from our review. As with P wave changes, prolonged PR intervals indicate remodeling of the atria (83). Future studies without comorbidities or confounders need to additionally include PR interval investigations.

Our review also considered several ventricular features, including QTc maximum, minimum, dispersion, duration, prolongation, peak, QTcF duration, QTfr Maximum, QT maximum, minimum, dispersion, duration, QRS duration, fragmented QRS, R wave amplitude, S wave amplitude, T wave maximum, minimum, dispersion, amplitude, TpTe, TpTe/QT ratio, and TpTe/QTc ratio. From a pathophysiological point of view, chronically elevated systemic BP leads to increased left ventricular pressure, which leads to left ventricular hypertrophy and interstitial fibrosis (84). These changes to the myocardium result in heterogenous changes to the action potential and repolarization duration, which in turn results in increased dispersion of ventricular ECG features (85).

Of the ventricular features, QT and QTc dispersion, QTc prolongation, and TpTe appeared promising, exhibiting significantly longer lengths with elevated BP. As with atrial features, there are numerous confounders and comorbidities that may affect ventricular features, such as age, sex, obesity, diabetes mellitus, obstructive sleep apnea, dynamic exercise, and electrolyte levels (86–89). When articles containing confounders were removed, the number of significant articles decreased and the results are summarized in Figure 3. There were still consistently a few significant findings for QTc dispersion, TpTe and TpTe/QTc ratio. These articles can be seen in Table 2. Note that QT and T wave variables have been reported to be associated both with hypertension and with hypertension and LVH (90). Interestingly, Bombelli et al. (91) found that TpTe did not predict the development of hypertension in a 10-year follow-up study. It was hypothesized that TpTe may occur from exposure to hypertension rather than preclude it (91). Fragmented QRS and R wave amplitude appeared promising as well, but after articles with comorbidities and confounders were removed, no other articles investigated these parameters. Some literature indicates that a significant presence of fragmented QRS is associated with more prolonged exposure to hypertension, more severe left ventricular diastolic dysfunction, or LVH (92, 93). R wave amplitude has been limitedly researched, but noted to not have a decrease in amplitude during exercise in hypertensive adolescent boys compared to normotensive adolescent boys (94).

When atrial and ventricular features are grouped, as in Figure 3, there is no clear evidence that atrial or ventricular features are significant or not significant with elevated BP when comorbidities and confounders are removed. This may indicate that specific ECG wave features may be more affected by elevated BP rather than groupings of atrial and ventricular features. However, the amount of data for each feature is too limited to draw specific conclusions.

The ECG wave parameter trend that was least promising for significant findings was related to minimal values, such as P wave minimum, QTc minimum, QT minimum, and T wave minimum. Of the 13 articles that mentioned minimum values, only one article found a significant difference in the minimum value.

In consideration of all the articles in our review, 16 articles had one or more comorbidities identified in their study population. Eleven of these articles had two or more conditions. Comorbidities such as diabetes (37, 76, 77), hyperlipidemia (95, 96), and thyroid dysfunction (97) may affect ECGs and obscure the true effect of hypertension on ECGs. Future studies comparing normotensive individuals to individuals with hypertension need comprehensive exclusion criteria to remove participants with comorbidities from the study population.

A further consideration is that 10 articles had significant differences between the age, sex or other variables between the different BP groups within the studies. There were also 15 articles which did not report if there were any significant differences between the different BP groups. Confounders such as differences in age (38), sex (38), high BMI (40, 41, 87), alcohol use (35) and smoking (34, 36) are known to affect BP. Future participant pools would benefit from eliminating differences between BP groups in these confounders and limiting alcohol and tobacco use amounts in the study population.

When looking in the results section the study population of the articles that showed insignificant results between blood pressure groups, were often younger, healthier, normotensive or recently diagnosed with hypertension. Note that all techniques used for measuring BP and collecting/analyzing ECG signals were similar between studies that showed significant or insignificant findings.

BP was measured by cuffed methods in every article in our review. The one article that also had an invasive, cuffless BP method, used it to confirm the BP categorization of their participants into hypertensive or normotensive groups. This leaves an opportunity for future studies to use invasive BP measurements to analyse ECG signals as they change with BP fluctuations.

### Special Populations

Of the three articles investigating pregnant populations, the most promising parameters were TpTe features. Interestingly, healthy pregnancies have been associated with increased heart rate, shortened AV conduction, prolonged QTc duration, and alterations in ventricular depolarization and repolarization (98, 99). Some of these changes do not resolve after delivery (98, 99). There is a theory that spatial changes in the thoracic cavity (similar to changes that occur with obesity) could cause changes in ECG parameters as well as hormonal changes, such as increased sympathetic activity and total body fluid (98). While there is currently not enough data about the differences between healthy pregnancies and pregnancies with hypertension, if we can develop a convenient ECG-only method of monitoring BP, we may be able to intervene early or prevent hypertensive crises in pregnancy.

There were two articles with pediatric populations that showed significant increases in P wave dispersion, but these two articles may have had a large overlap in their study populations as they both included participants from the same project pool of participants (66, 67). In addition, one article included a participant population that had 16.8% with obesity (66), and the other article did not disclose the BMIs of their pediatric population (67). Obesity has been associated with increased P wave dispersion and duration in adult populations (75, 78). In a pediatric population, obesity and status as a pediatric athlete were associated with increased QTc and QT dispersion (68, 100). It is unclear in the limited pediatric articles if P wave or other ECG parameters change with elevated BP in pediatric patients.

### ECG Parameter Estimating

There was also a variety of manual and computer software methods for estimating ECG parameters. Manual methods used one, two, three or an unspecified number of individuals to estimate ECG parameters. These individuals were described in various ways such as cardiologists, investigators, general practitioners, experts, observers, or were not described. This wide variety is concerning as it is known that ECG interpretations and the frequency of interpretation errors can differ depending on the training level of the ECG reader (101–103). Interestingly, confirmation biases can occur with ECG interpretations when clinical details about the patient are included (103). Of the 26 articles that mentioned using manual methods, only 12 reported whether the individuals were blinded to the participants' clinical status. This highlights that it is important to consider who will be estimating the ECG parameters, how much knowledge they have of the participants, their training level and how many individuals are needed to minimize biases. The different computer software packages employed were either used alone or a mix of computer software and manual reviewing was used. Interestingly, almost all the computer methods used different software and only two studies alluded to why their software was chosen. It would be valuable to know why certain software package were chosen by the investigators to help evaluate the reliability of the ECG results. For example, Hassing et al. reported that they used an algorithm which has been previously used in studies and was known to meet all the International Electrotechnical Commission requirements for amplitude estimation (13). Vaidean et al. noted their ECG analysis software was common in primary health care (83). Three articles did not describe how their ECG parameters were estimated. Consistent reporting and careful consideration of the manual and/or computer methods used in future articles is needed.

### Considerations for Portable, ECG-Only BP Monitoring

There is a need for 24-h continuous, accurate, and non-intrusive monitoring of BP for the general population. Some articles show that it is possible to integrate lightweight, portable patient monitoring devices into daily life (104, 105). Increasing the usability of ECGs will make health surveillance more accessible in rural and resource-limited settings (106).

The challenge is deciding whether there are ECG parameters for which there is meaningful data indicating that they can identify hypertension early in its disease process. Looking at articles without comorbidities or confounders, four ECG wave parameters had more than one article finding them significantly difference with increases in BP. P wave dispersion was the only atrial features which appeared promising in this review. Three ventricular features appeared promising involving QTc dispersion, TpTe and TpTe/QTc ratio. However, there is limited data as most articles included comorbidities or confounders that may have affected the results. Further articles investigating atrial and ventricular feature changes between normotension and hypertension in otherwise healthy populations are needed. There can also be investigations into machine learning with ECG signals to consider whether meaningful BP data can be obtained from ECG wave morphology (26–28).

**Figure 4:**
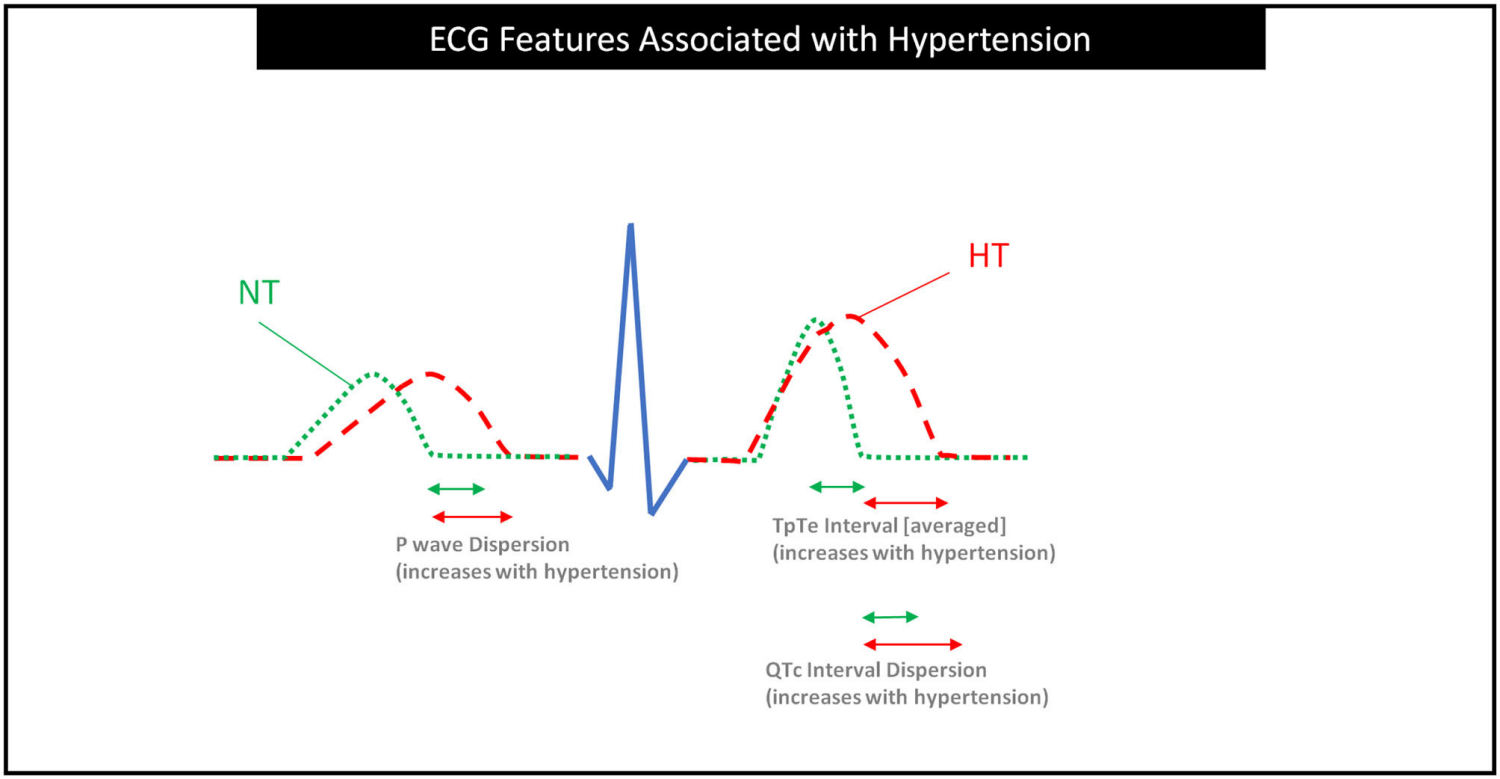
ECG features associated with hypertension based on this study. It is clear that P wave dispersion, TpTe interval, and QTc interval dispersion are increasing in subjects with hypertension. Dispersions are calculated from the difference between the maximum and minimum lengths of the feature. This image shows the normotensive individual as a minimum length and the hypertensive individual as a maximum length to illustrate the longer dispersions in hypertensive individuals. The green arrows are approximated shorter as the normotensive dispersions are significantly shorter from the results of this review. NT, Normotensive; HT, Hypertensive.

To move the ECG technology forward we sought to summarize the promising ECG features that are associated with hypertension, as shown in Table 1. This was carried out counting in the ECG features that were consistently reported to be correlated with BP. The visual representations of the promising ECG features are shown in Figure 4. It can be seen that P wave dispersion is the difference between the longest and shortest P wave durations (13, 67). This difference has been seen to increase in those with hypertension compared to those with normotension. TpTe is the length of the interval from the peak of the T wave to the end of the T wave (13, 56). Its duration is an average of several TpTe estimations. TpTe has also been seen to increase in length in those with hypertension compared to those with normotension. Thirdly, QTc dispersion is the difference between the longest and shortest QTc durations and it increases in length in those with hypertension. Finally, TpTe/QTc duration was also listed in Table 1 as increasing with hypertensive individuals. It was not included in Figure 4 as it is a calculation from ECG wave morphology than a direct ECG wave morphology to be visualized. These four ECG wave features appear the most promising in identifying hypertensive individuals and would be promising features for future research.

Another consideration related to bringing ECGs into daily life is the burden of using multiple leads to collect 12-lead ECG data. There is limited data regarding the leads on which it may be important to focus for monitoring for ECG changes. There are discussions about whether using six leads instead of a full 12-lead ECG would decrease the accuracy of QTc dispersion estimation (99). The one article in our review which used only one lead (lead III) for QT and QTc estimation did not find significant ECG changes with elevated BPs (31). Ten other articles in our review described specific single lead results. However, with the number of different ECG wave parameters estimated and the variance in which single leads were reported, further investigations describing all the leads and their specific findings are needed.

Devices with limited lead approaches are showing potential. Smartphones with an additional small handheld device have the potential to identify atrial fibrillation using a single ECG lead approach (107). Smartwatches also can capture multiple types of single lead estimations and possibly provide enough data to identify ischemic changes such as ST elevations (108). Research commenting on the specific leads used and comparing leads would be beneficial for the development of convenient, daily-life BP monitoring.

A third consideration is the type of activity in which a person is involved while being monitored, which may alter the ECG results. For example, Bhandari et al. found that non-obese young men exhibited significant changes in heart rate, BP, and QTc interval after dynamic exercise (88). It would be important for future studies to investigate if continuous BP monitoring with ECGs during dynamic activities is relevant for diagnosing hypertension, or if BP readings during dynamic exercise need to be left out.

Finally, it is important to investigate the setting of ECG parameter estimation. For example, increases in heart rate have been correlated with cardiovascular mortality when measured in a clinical setting (19). However, when heart rate is taken in ambulatory settings, the correlation is lost, possibly due to community confounding factors or a unique attribute of the office setting, such as sympathetic activation (19). Conversely, BP measurements taken at home rather than in an office setting are more correlated with LVH (109). This underlines that it is important to not only consider ECG parameter estimations in clinical settings but also to investigate ECGs in ambulatory settings to determine whether there are any differences in results.

One limitation of this review is that there may be more ECG wave parameters in the literature that were not identified by our original search terms. We chose to filter articles by the main ECG wave parameters to reduce and focus the number of articles for the hand search. We used a preliminary literature search to identify our listed ECG wave parameters. A further limitation common to research reviews is that articles may only be published or report findings if there was significance.

Another limitation across the included articles was inconsistent reporting of comorbidities or confounders. Therefore, some articles without any reported comorbidities or confounders, such as obesity, smoking habit, or diabetes mellitus, may have had a comorbidity or confounder that was not identified. This is considering that for example in the world, 8.3% of adults have diabetes (110), 12.5% of adults are obese (111), and 15.2% of adults smoke tobacco daily (112). There was also inconsistent reporting of the average duration of hypertension diagnosis and whether medications were used. Finally, there were variations in participants' age and sex, which may have influenced ECG parameter results (38). These factors would add a degree of uncertainty as they would in most research studies.

## Recommendations

We found 36 articles for our literature review, 35 articles were included for our analysis, and between them they studied a wide variety of ECG wave parameter changes with BP changes. Currently, there is no well-supported consensus regarding these parameters. Therefore, our ability to develop recommendations for specific ECG parameters is limited. We suggest ways in which future research can address these concerns.

Based on our literature review of ECG-only BP measurement and hypertension identification, we recommend the following:

1. Continuing research needs to be conducted with larger participant sample sizes, clear exclusion and inclusion criteria, and the use of young, healthy subjects to establish a more robust relationship between ECG features and BP.
2. Future research studies need to include hypertensive, prehypertensive, and normotensive populations or the use of subgroups with a fixed range of BP (e.g., intervals of 5 mmHg for systolic, diastolic, or both) increases to clarify if there is a linear trend in terms of BP increase.
3. Reporting the duration of hypertension and prehypertension diagnoses among study participants will help clarify whether ECG parameters are dependent on the amount of time a participant has had hypertension or prehypertension.
4. When researching ECG parameters for BP, there is an opportunity to collect and report a wide variety of ECG parameter data to increase the amount of data available to analyze the significance of the various possible ECG parameters.
5. Reporting if ECG parameters are estimated using manual methods or computer software methods would be important to clarify if different methods are producing different results or biases. Including the training background of the manual estimaters (i.e., cardiologist, general practitioner, etc.), the number of individuals involved, the individuals' involvement/understanding of the study and participants, the computer software used and the reason the computer software was chosen would be valuable information for considering the reliability of reported ECG results.
6. The current understanding of the pathophysiology underlying ECG wave morphology changes is cardiac tissue adaptation from chronic exposure to high BP. There is a need for research studies that follow participants over time to investigate how ECG parameters can change and how long it takes for them to show change. These findings can determine whether ECG changes occur before or after hypertension is diagnosed and guide how frequently ECG-only monitoring could occur.
7. BP prediction models using a combination of ECG parameters need to be explored. This would give us the opportunity to predict who may need early interventions to prevent hypertension and its disease progression. Artificial intelligence can play a major role here.
8. There is an opportunity to compare systolic and diastolic BPs to see if there are significantly different ECG findings between them. We found only one study in our review which looked at SBP and DBP separately (13). This can further our understanding if ECGs can differentiate normotensive vs. elevated BPs for both systolic and diastolic settings.
9. Consistent listing or exclusion of ECG confounders, such as hypertension medication, smoking habit, alcohol use, and BMI can help with the interpretation of significant ECG results.
10. Future research needs to consider investigating pregnant and pediatric populations. If hypertension can be identified early in life or during significant life events such as pregnancy, intervention can be implemented early and disease progression can be slowed or prevented.
11. Investigations need to compare specific ECG changes due to hypertension to changes to the heart from athletic activities. We are aware of one study that did not find PQ, QRS, or QT parameters to be different between these groups (113).
12. Limiting the number of ECG electrodes used for data collection to 1-, 3-, or 6-lead ECGs (compared to a full standard 12-lead ECG) would lead to an understanding if we can obtain reliable data from less leads. With less leads, more convenient and portable ECG signal collection devices may be used to monitor BP. Future articles reporting the results from all 12 leads would provide a way to investigate if one or a few leads would be more reliable to focus on for monitoring BP.
13. In future studies using ECG parameters to predicting BP, it will be important to provide a thorough analysis including the accuracy of identifying or predicting hypertension, the agreement between the BP measurement using a gold standard and estimated BP utilizing ECG signal, and the correlation between ECG parameters and BP measurement.

## Conclusions

This review summarized the literature on the use of only ECG signals to monitor blood pressure or identify hypertension from January 1, 2010, to January 1, 2020. In the 35 analyzed articles, P wave dispersion, QT and QTc dispersion, QTc prolongation, fragmented QRS, R wave amplitude, and TpTe were found to be promising as they were significantly associated with higher systolic or diastolic blood pressure. However, there were limited articles for each ECG wave parameter. When articles with comorbidities and confounders for ECG changes were removed, one atrial feature of P wave dispersion was often significant with higher BPs. Three ventricular features of QTc dispersion, TpTe interval, and TpTe/QTc ratio were found to be promising as well. However, most articles without comorbidities or confounders were in pediatric or pregnant populations. With this wide variety of participant characteristics, further data is needed before conclusions about the usefulness of ECG wave morphology for monitoring BP can be made.

ECGs have the potential to provide a convenient and long-term way to monitor BP in the future with the support of further research and the growth of smart personal devices. We have made 13 recommendations for future ECG-only BP monitoring studies. It is important for further research to include pediatric and pregnant populations as these populations would benefit from early monitoring and intervention.

## Data Availability Statement

The raw data supporting the conclusions of this article will be made available by the authors, without undue reservation.

## Author Contributions

ME designed the study and led the investigation and drafted the manuscript for submission with revisions and feedback from the contributing authors. KB screened all full-text articles. KB, GC, HL, HG, JA, DA, RW, NH, W-SC, CM, NL, AA, RF, and ME conceived the study, provided directions, feedback, and revised the manuscript. All authors approved the final manuscript.

## Conflict of Interest

The authors declare that the research was conducted in the absence of any commercial or financial relationships that could be construed as a potential conflict of interest.

## Abbreviations

AC Filter: Alternating Current Filter
AV Conduction: Atrioventricular Conduction
BMI: Body Mass Index
BP: Blood Pressure
DBP: Diastolic Blood Pressure
ECG or EKG: Electrocardiogram
HRV: Heart Rate Variability
HT: Hypertensive
Hz: Hertz
LAE: Left Atrial Enlargement
LVDD: Left Ventricular Diastolic Dysfunction
LVH: Left Ventricle Hypertrophy
mmHg: Millimeter of Mercury
ms: Milliseconds
NR: Not Reported
NS: Not Significant
NT: Normotensive
PHT: Prehypertensive
PIHT: Pregnancy-Induced Hypertension
PPG: Photoplethysmography
QTc: QT interval corrected for heart rate using Bazett's formula: QT/√R –R (11), or Hodges formula: [QTc = QT + 1. 75 (heart rate – 60)] (12)
QTd: QT Interval Dispersion
SBP: Systolic Blood Pressure
STD: Standard Deviation
TpTe: Length between peak of T wave to the end of the T wave (13).
