# Supplementary material for: Assessment of Hypertension Using Clinical Electrocardiogram Features: A First-Ever Review
Source: Front Med (Lausanne). 2020 Dec 4;7:583331. doi: 10.3389/fmed.2020.583331 (PMC7746856; doi:10.3389/fmed.2020.583331)
Supplement: Supplementary file 1 [file Table_1.pdf]

APPENDIX 1

<u>ECG search terms:</u> "ECG OR EKG OR Electrocardiogra*"

<u>BP search terms:</u> "Blood pressure monitor* OR blood pressure measur* OR blood pressure read* OR blood pressure estimat* OR blood pressure assess* OR continuous blood pressure OR non-invasive blood pressure OR noninvasive blood pressure OR non invasive blood pressure OR invasive blood pressure OR wearable blood pressure OR cuffless blood pressure OR cuff-less blood pressure OR cuffless blood pressure OR BP monitor* OR BP measur* OR BP read* OR BP estimat* OR BP assess* OR continuous BP OR non-invasive BP OR noninvasive BP OR non invasive BP OR invasive BP OR wearable BP OR cuffless BP OR cuff-less BP OR cuff less BP OR Hypertension"

<u>Excluded Reviews, Case studies or commentaries terms: </u>"Systematic Review* OR Comment* OR Case Report*"

<u>Excluded Non-English articles:</u> Applied "English" filter

<u>Excluded photoplethysmography technology terms</u>: "PPG OR photoplethysmogra* OR plethysmograp* OR PTT OR pulse transit time OR PAT OR pulse arrival time"

<u>ECG wave morphology related words</u>: "ECG marker* OR ECG signal* OR Segment* OR P wave OR R wave OR T wave OR RR interval OR Fragmented QRS OR J point OR ST depression OR interatrial block OR PR interval OR QT interval OR QTc interval OR S wave OR TpTe OR Tp-e OR PR OR QRS OR QTc OR QTcF OR QT OR ST"

APPENDIX 2

Final search terms in Legacy PubMed, March 27th, 2020:

"((((((((((((ECG[Title/Abstract] OR EKG[Title/Abstract] OR Electrocardiogra*[Title/Abstract])) AND (Blood pressure monitor*[Title/Abstract] OR blood pressure measur*[Title/Abstract] OR blood pressure read*[Title/Abstract] OR blood pressure estimat*[Title/Abstract] OR blood pressure assess*[Title/Abstract] OR continuous blood pressure[Title/Abstract] OR non-invasive blood pressure[Title/Abstract] OR noninvasive blood pressure[Title/Abstract] OR non invasive blood pressure[Title/Abstract] OR invasive blood pressure[Title/Abstract] OR wearable blood pressure[Title/Abstract] OR cuffless blood pressure[Title/Abstract] OR cuff-less blood pressure[Title/Abstract] OR cuffless blood pressure[Title/Abstract] OR BP monitor*[Title/Abstract] OR BP

measur*[Title/Abstract] OR BP read*[Title/Abstract] OR BP estimat*[Title/Abstract] OR BP assess*[Title/Abstract] OR continuous BP[Title/Abstract] OR non-invasive BP[Title/Abstract] OR noninvasive BP[Title/Abstract] OR non invasive BP[Title/Abstract] OR invasive BP[Title/Abstract] OR wearable BP[Title/Abstract] OR cuffless BP[Title/Abstract] OR cuff-less BP[Title/Abstract] OR cuff less BP[Title/Abstract] OR Hypertension[Title/Abstract])) AND ("2010/01/01"[PDat] : "2020/01/01"[PDat]))) NOT (Systematic Review*[Title/Abstract] OR Comment*[Title/Abstract] OR Case Report*[Title/Abstract])) AND ("2010/01/01"[PDat] : "2020/01/01"[PDat]) AND English[lang])) NOT (PPG[Title/Abstract] OR photoplethysmogra*[Title/Abstract] OR plethysmograp*[Title/Abstract] OR PTT[Title/Abstract] OR pulse transit time[Title/Abstract] OR PAT[Title/Abstract] OR pulse arrival time[Title/Abstract])) AND ("2010/01/01"[PDat] : "2020/01/01"[PDat]) AND English[lang])) AND (ECG marker*[Title/Abstract] OR ECG signal*[Title/Abstract] OR Segment*[Title/Abstract] OR P wave[Title/Abstract] OR R wave[Title/Abstract] OR T wave[Title/Abstract] OR RR interval[Title/Abstract] OR Fragmented QRS[Title/Abstract] OR QRS[Title/Abstract] OR J point[Title/Abstract] OR ST depression[Title/Abstract] OR interatrial block[Title/Abstract] OR PR interval[Title/Abstract] OR QT interval[Title/Abstract] OR QTc interval[Title/Abstract] OR S wave[Title/Abstract] OR TpTe[Title/Abstract] OR QTc[Title/Abstract] OR QTcF[Title/Abstract] OR QT[Title/Abstract] OR PR[Title/Abstract] OR Tp-e[Title/Abstract] OR ST[Title/Abstract] OR RR[Title/Abstract])) AND ("2010/01/01"[PDat] : "2020/01/01"[PDat]) AND English[lang]) Filters: Publication date from 2010/01/01 to 2020/01/01; English”

APPENDIX 3

List of all 36 studies selected in this review. For green shaded studies, no comorbidities or confounders were listed, and for grey shaded studies, there were comorbidities present or confounders. NT = normotensive, PHT = prehypertensive, HT = hypertensive, PIHT = pregnancy-induced hypertension, BMI = body mass index. $\bar{x}$ = mean of variable x reported by the article, where x can be BMI or age. $\tilde{x}$ = standard deviation reported by the article, where x can be BMI or age. $\bar{x}_p$ = calculated pooled mean of variable x, where x can be BMI or age. $\tilde{x}_p$ = calculated pooled standard deviation of variable x, where x can be BMI or age. NR = Not reported. * = Significant difference with an increase in BP. ** = unclear if significant results are missing for this parameter

**Study**	**Sample Size**	**NT/PHT/PIHT/ HT**	**Comorbidities/Possible BP Confounders/Notes**	**BP Categorizations**	**Gold Standard for BP Measurement**	**Variables of Significance Between BP groups**	**ECG Parameter Estimated**	**ECG Leads Used**	**ECG Feature Estimation/Computation Method**

**Gazi et al. (2016)**[1]	41	NT: 24 (58.5%)<br>PHT: 0<br>PIHT: 17 (41.5%)<br>HT: 0	No listed comorbidities<br>Excluded multiple comorbidities<br>Pregnancy<br><br>$\overline{\text{Age}}_p$ = 28.2 years<br>$\widetilde{\text{Age}}_p$ = 5.7 years<br><br>100.0% Female<br><br>No BMIs reported	<u>PIHT:</u><br>SBP > 140 mmHg and/or DBP > 90 mmHg in two measurements or more after 20 weeks of gestation<br><br><u>NT:</u><br>NR	<u>Cuffed Mercury Sphygmomanometer:</u><br>Details NR	No significant differences in reported variables	P wave Maximum<br>P wave Minimum<br>P wave Dispersion<br><br>QTc Maximum<br>QTc Minimum<br>QT Dispersion<br>QT Maximum*<br>QT Minimum<br><br>TpTe*<br>TpTe/QT<br>TpTe/QTc*	12-lead	Manual (one cardiologist, blinded to clinical statuses)
**Kirbas et al. (2016)**[2]	96	NT: 32 (33.3%)<br>PHT: 0<br>PIHT: 64 (66.6%)<br>HT: 0	Pregnancy<br>Preeclampsia<br>Excluded multiple comorbidities<br><br>$\overline{\text{Age}}_p$ = 27.6 years<br>$\widetilde{\text{Age}}_p$ = 5.1 years<br><br>100.0% Female<br><br>$\overline{\text{BMI}}_p$ = 29.3 kg/m^2<br>$\widetilde{\text{BMI}}_p$ = 3.5 kg/m^2	<u>Mild PIHT:</u><br>SBP ≥ 140 mmHg or DBP ≥ 90 mmHg and 300 mg/dL proteinuria in 24-hour urine collection or systemic disease symptoms after 20 weeks of gestation. HT plus systemic disease (cerebral disturbances, visual disturbances, pulmonary edema, renal failure, increased liver transaminases, thrombocytopenia) instead of proteinuria also qualified.<br><br><u>Severe PIHT:</u><br>SBP ≥ 160 mmHg or DBP ≥ 110 mmHg at two	<u>Gold Standard Method NR:</u><br>Details NR	No significant differences in reported variables	QTc Maximum*<br>QTc Minimum<br>QTc Dispersion*<br>QTc Duration*<br><br>TpTe, Lead II, V2, V5*<br>TpTe/QTc*	12-lead	Manual (two cardiologists, blinded to clinical statuses)

				measurements at least six hours apart, pulmonary edema, thrombocytopenia, elevated hepatic transaminase, epigastric pain, visual impairment, headache or elevated serum creatinine.<br><br><u>NT:</u><br>NR					
**Tanindi et al. (2015)[3]**	84	NT: 37 (44.0%)<br>PHT: 47 (56.0%)<br>PIHT: 0<br>HT: 0	No listed comorbidities<br>Excluded multiple comorbidities<br><br>$\overline{\text{Age}}_p$ = 51.5 years<br>$\widetilde{\text{Age}}_p$ = 13.1 years<br><br>56.0% Female<br><br>$\overline{\text{BMI}}_p$ = 26.3 kg/m^2<br>$\widetilde{\text{BMI}}_p$ = 2.9 kg/m^2	<u>PHT:</u><br>SBP = 120–139 mmHg and DBP 80–89 mmHg.<br><br><u>NT:</u><br>NR	<u>Cuffed Mercury Sphygmomanometer:</u><br>• *# of BPs:* 2 in first visit & repeated in a second visit<br>• *Interval between measurements:* 10 minutes<br>• *Was average or maximum used for final BP value:* Average<br>• *Rest duration prior to BP measuring:* 5 minutes	No significant differences in reported variables	QTc Maximum<br>QTc Minimum<br>QTc Dispersion*<br><br>TpTe*<br>TpTe/QT*	12-lead	Manual (two cardiologists, NR if blinded to clinical statuses)
**Chávez et al. (2014)[4]**	656	NT: 394 (60.0%)<br>PHT: 199 (30.3%)<br>PIHT: 0<br>HT: 63 (9.7%)	Children 8 – 11 years old (Tanner stage 1)<br>Excluded multiple comorbidities<br><br>50.3% Female<br><br>No BMIs reported<br>Obese (16.8%)	<u>HT:</u> BP > 95th percentile<br><br><u>PHT:</u> BP 90–95th percentile<br><br><u>NT:</u> BP < 90th percentile	<u>Cuffed Sphygmomanometer</u>:<br>• *# of BPs:* 4 on different days<br>• *Interval between measurements:* NR	Article did not compare between BP groups	P wave Dispersion*	12-lead	Manual (three experts, NR if blinded to clinical statuses)

				Percentiles were specific for age, sex and height.	• *Was average or maximum used for final BP value:* NR<br>• *Rest duration prior to BP measuring:* 10 minutes				
**Kirbas et al. (2014)[5]**	88	NT: 30 (34.1%)<br>PHT: 0<br>PIHT: 58 (65.9%)<br>HT: 0	Pregnancy<br>Preeclampsia<br>Excluded multiple comorbidities<br><br>$\overline{\text{Age}}_p$ = 28.0 years<br>$\widetilde{\text{Age}}_p$ = 5.3 years<br><br>100.0% Female<br><br>$\overline{\text{BMI}}_p = 29.3\ kg/m^2$<br>$\widetilde{\text{BMI}}_p = 2.8\ kg/m^2$	<u>Mild PIHT:</u><br>SBP ≥ 140 mmHg or DBP ≥ 90 mmHg at two times at 4 four or more hours apart after 20 weeks of gestation, plus a positive dipstick for proteinuria or ≥ 300 mg/dL proteinuria in 24-hour urine collection.<br><br><u>Severe PIHT:</u><br>SBP ≥ 160 mmHg or DBP ≥ 110 mmHg at two times at 4 four or more hours, or one or more of: severe right hypochondriac pain, pulmonary edema, visual or cerebral symptoms, liver transaminases twice normal, thrombocytopenia, renal insufficiency.<br><br><u>NT:</u><br>NR	<u>Gold Standard Method NR:</u><br>Details NR	No significant differences in reported variables	P wave Maximum<br>P wave Minimum*<br>P wave Dispersion*	12-lead	Manual (one principle investigator, blinded to clinical statuses)

**Chávez et al. (2013)[6]**	515	NT: 333 (64.7%)<br>PHT: 156 (30.3%)<br>PIHT: 0<br>HT: 26 (5.0%)	Children 8 – 11 years old (Tanner stage 1)<br>Excluded multiple comorbidities<br><br>No mean age, sex percentages or BMIs reported	<u>HT:</u> BP > 95th percentile<br><br><u>PHT:</u> BP 90–95th percentile<br><br><u>NT:</u> BP < 90th percentile<br><br>Percentiles were specific for age, sex and height.	<u>Cuffed Oscillometric Sphygmomanometer</u>:<br>• *# of BPs:* 4 on different days<br>• *Interval between measurements:* NR<br>• *Was average or maximum used for final BP value:* Average<br>• *Rest duration prior to BP measuring:* 10 minutes	Article did not compare between BP groups	P wave Dispersion*	12-lead	Method NR
**Chávez et al. (2013)[7]**	515								
**Anigbogu et al. (2012)[8]**	78	NT: 39 (50.0%)<br>PHT: 0<br>PHIT: 0<br>HT: 39 (50.0%)	Excluded any disease conditions aside from hypertension.<br>Excluded significant alcohol use, smoking, and medications which affected cardiovascular function.<br><br>No mean age reported<br><br>Sex not described<br><br>No BMIs reported	<u>HT:</u><br>BP > 140/90 mmHg on two readings.<br><br><u>NT:</u><br>NR	<u>Cuffed Sphygmomanometer</u>:<br>• *# of BPs:* ≥2<br>• *Interval between measurements:* NR<br>• *Was average or maximum used for final BP value:* NR<br>• *Rest duration prior to BP measuring:* NR	Article did not compare between BP groups	P wave Amplitude**<br><br>PR Interval Duration<br><br>QRS Duration*<br>QRS Amplitude**<br><br>QT Interval Duration<br><br>ST Interval Duration<br><br>T wave Amplitude**	12-lead	Method NR
**Yildirim et al. (2012)[9]**	48	NT: 24 (50.0%)<br>PHT: 0<br>PHIT: 0<br>HT: 24 (50.0%)	Excluded multiple comorbidities<br><br>$\overline{Age}_p$ = 44.4 years<br>$\widetilde{Age}_p$ = 9.7 years	<u>HT:</u><br>SBP ≥ 140 mmHg or DBP ≥ 90 mmHg or average ambulatory daytime	<u>Cuffed Mercury Sphygmomanometer</u>:<br>• *# of BPs:* 3	No significant differences in reported variables	P wave Maximum*<br>P wave Minimum<br>P wave Dispersion*	12-lead	Manual (one cardiologist, blinded to

			68.8% Female<br><br>$\overline{BMI}_p$ = 30.0 kg/m^2<br>$\widetilde{BMI}_p$ = 3.0 kg/m^2	BP ≥ 135/85 and no HT treatment.<br><br><u>NT:</u><br>SBP < 140 mmHg and DBP < 90 mmHg and average ambulatory daytime BP < 135/85 and no HT treatment.	• *Interval between measurements:* NR<br>• *Was average or maximum used for final BP value:* Average<br>• *Rest duration prior to BP measuring:* 5 minutes<br><br>Plus<br><br><u>Cuffed 24-hour Non-Invasive Ambulatory Oscillometric Device:</u><br>• *Interval between measurements:* NR<br>• *Was average or maximum used for final BP value:* Average<br>• *Repeated if over 20% of the readings were invalid:* NR. They were considered invalid.				clinical statuses)
**Zhao et al. (2010)[10]**	121<br><br>(83 without LVH)	NT: 42 (50.6%)<br>PHT: 0<br>PHIT: 0<br>HT: 41 (49.4%)	No listed comorbidities<br>Excluded multiple comorbidities<br><br>$\overline{Age}_p$ = 57.3 years<br>$\widetilde{Age}_p$ = 12.0 years<br><br>44.6% Female<br><br>No BMIs reported	<u>HT:</u><br>According to JNC7 criteria:<br>SBP ≥ 140 mmHg or DBP ≥ 90 mmHg[11]<br><br><u>NT:</u><br>NR	<u>Gold Standard Method NR:</u><br>Details NR	No significant differences in reported variables	QTc Peak, Leads V4 – V6<br>QTc Duration, Leads V4 – V6<br><br>TpTe(c), Leads V4 – V6<br>TpTe(c)/QTc, Leads V4 – V6	12-lead	Manual (one physician, NR if blinded to clinical statuses)

**Hassing et al. (2020)[12]**	1,449	NT: 1,197 (82.6%)<br>PHT: 252 (17.4%)<br>(by systolic BP)<br>PIHT: 0<br>HT: 0	After medical screening, only healthy participants included.<br><br>$\overline{\text{Age}}$ = 22.7 years<br>$\widetilde{\text{Age}}$ = 3.0 years<br><br>26.3% Female<br><br>$\overline{\text{BMI}}_p$ for SBP: 22.7 kg/m^2<br>$\widetilde{\text{BMI}}_p$ for SBP: 2.5 kg/m^2	<u>Five SBP groups:</u><br><br>90–99 mmHg<br>100–109 mmHg<br>110–129 mmHg<br>120–129 mmHg<br>130–139 mmHg*<br><br>*The article considered this group high normal (PHT)	<u>Cuffed Sphygmomanometer</u>:<br>• *# of BPs:* NR<br>• *Interval between measurements:* NR<br>• *Was average or maximum used for final BP value:* NR<br>• *Rest duration prior to BP measuring:* 5 minutes	BMI for SBP<br>($p < 0.05$)	P wave Maximum<br>P wave Dispersion<br>P wave Area, Lead V1*<br><br>PR Interval Duration<br><br>QTcF Duration*<br><br>QRS Duration*<br>R wave Axis<br><br>T wave Maximum*<br>T wave Minimum<br>T wave Dispersion<br><br>TpTe<br><br>Ventricular Activation Time in Lead V6*<br>J point T peak Duration*	12-lead	Computed (Marquette 12SL algorithm)
**Bekar et al. (2019)[13]**	353	NT: 153 (43.3%)<br>PHT: 0<br>PHIT: 0<br>HT: 200 (56.7%)	Hyperlipidemia (35.9%)<br>Smoking (14.6%)<br>Excluded multiple comorbidities<br><br>$\overline{\text{Age}}_p$ = 51.6 years<br>$\widetilde{\text{Age}}_p$ = 7.3 years<br><br>63.7% Female<br><br>$\overline{\text{BMI}}_p$ = 30.9 kg/m^2<br>$\widetilde{\text{BMI}}_p$ = 5.1 kg/m^2	<u>HT:</u><br>BP ≥ 140/90 mmHg or ambulatory 24-hour BP averaged ≥130/80 or daytime ambulatory averaged ≥ 135/85 or nighttime ambulatory BP averaged ≥120/70 or diagnosed previously with HT and taking HT medication for at least two months.	<u>Cuffed Sphygmomanometer</u>:<br>• *# of BPs:* ≥ 2<br>• *Interval between measurements:* NR<br>• *Was average or maximum used for final BP value:* NR<br>• *Rest duration prior to BP measuring:* 10 minutes	BMI<br>($p < 0.001$)	Fragmented QRS*	12-lead	Manual (two cardiologists, blinded to clinical statuses)

				<u>NT:</u><br>NR	Plus<br><br><u>24-hour Ambulatory BP Measurements:</u><br>Further details NR.				
**Dzikowicz & Carey (2019)[14]**	77	NT: 23 (29.9%)<br>PHT: 29 (37.7%)<br>PIHT: 0<br>HT: 25 (32.4%)	Sleep Apnea (3.9%)<br>Left Ventricular Hypertrophy (2.6%)<br>Smoking (13.0%)<br><br>$\overline{Age}$ = 43.4 years<br>$\widetilde{Age}$ = 7.8 years<br><br>3.9% Female<br><br>$\overline{BMI}$ = 29.4 kg/m²<br>$\widetilde{BMI}$ = 4.4 kg/m²	<u>HT*:</u><br>BP ≥ 140/90 mmHg<br><br><u>PHT*:</u><br>SBP = 130–139 mmHg or DBP = 80–89 mmHg<br><br><u>NT*:</u><br>SBP < 130 mmHg and DBP < 80 mmHg<br><br>*Article used 2017 American Heart Association BP guidelines[15]	<u>Gold Standard Method NR:</u><br>• *# of BPs:* 2<br>• *Interval between measurements:* 5 minutes<br>• *Was average or maximum used for final BP value:* Average<br>• *Rest duration prior to BP measuring:* 5 minutes	Article did not compare between BP groups	QRS Duration	12-lead	Mixed<br><br>Computed (H-Scribe 4 software & ELI LINK program)<br><br>and<br><br>Manual review (one reviewer, blinded to clinical statuses)
**Eyuboglu et al. (2019)[16]**	216	NT: 61 (28.2%)<br>PHT: 155 (71.8%)<br>PIHT: 0<br>HT: 0	Diabetes Mellitus (8.3%)<br>Smoking (17.6%)<br>Excluded HT and multiple cardiovascular diseases.<br><br>$\overline{Age}$ = 50.5 years<br>$\widetilde{Age}$ = 4.3 years<br><br>45.8% Female<br><br>No BMIs reported	<u>HT*:</u><br>Ambulatory 24-hour BP average SBP ≥ 130 mmHg and/or DBP ≥ 80 mmHg and/or daytime average is SBP ≥ 135 mmHg and DBP ≥ 85 mmHg.<br><br><u>PHT:</u><br>SBP = 120–139 and/or DBP = 80–89 and was not HT as per above during	<u>Gold Standard Method NR:</u><br>Details NR<br><br>Plus<br><br><u>Cuffed 24-hour Ambulatory BP with Oscillometric Device:</u><br>• *Interval between measurements:* 30 minutes<br>• *Was average or maximum used for final BP value:* Average	No significant differences in reported variables	Fragmented QRS*	12-lead	Manual (two cardiologists, blinded to clinical statuses)

				ambulatory BP measurement.<br><br>NT:<br>Ambulatory 24-hour BP average SBP < 120 mmHg and DBP < 80 mmHg<br><br>*Study excluded this group.	• *Repeated if over 20% of the readings were invalid:*<br>Yes				
**Sun et al. (2019)**[17]	11,264	NT: 3,111 (27.6%)<br>PHT: 2,406 (21.4%)<br>PIHT: 0<br>HT: 5,747 (51.0%)	Diabetes Mellitus (10.3%)<br>Smoking (35.2%)<br>Alcohol use (22.3%)<br>Anti-HT medication (16.2%)<br>Anti-arrhythmia medication (0.6%)<br>Echocardiogram abnormalities<br>History of MI (1.1%)<br>History of HF (0.8%)<br>Mitral stenosis/regurgitation (1.6%)<br>Excluded multiple cardiovascular diseases<br><br>$\overline{Age}_p$ = 53.7 years<br>$\widetilde{Age}_p$ = 10.6 years<br><br>54.5% Female<br><br>$\overline{BMI}_p$ = 24.8 kg/m^2<br>$\widetilde{BMI}_p$ = 3.7 kg/m^2	HT:<br>SBP ≥ 140 mmHg and/or DBP ≥ 90 mmHg and/or taking HT medication<br><br>NT:<br>NR	Cuffed Automatic Electronic Sphygmomanometer:<br>• *# of BPs:*<br>3<br>• *Interval between measurements:*<br>2 minutes<br>• *Was average or maximum used for final BP value:*<br>Average<br>• *Rest duration prior to BP measuring:*<br>5 minutes	Article did not compare between BP groups	P wave Prolongation*	12-lead	Computed (MUSE Cardiology Information System)

**Sun et al. (2019)[18]**	10,553	NT: 5192 (49.2%)<br>PHT: 0<br>PHIT: 0<br>HT: 5,341 (50.8%)	Smoking (35.2%)<br>Alcohol Use (22.2%)<br>Heart Disease History (9.8%)<br>Take any medication (53.2%)<br>Excluded multiple comorbidities<br><br>$\overline{\text{Age}}_p$ = 53.7 years<br>$\widetilde{\text{Age}}_p$ = 10.4 years<br><br>54.8% Female<br><br>$\overline{\text{BMI}}_p$ = 24.8 kg/m^2<br>$\widetilde{\text{BMI}}_p$ = 3.6 kg/m^2	<u>HT:</u><br>SBP ≥ 140 mmHg and/or DBP ≥ 90 mmHg and/or taking HT medication<br><br><u>NT:</u><br>NR	<u>Cuffed Automatic Electronic Sphygmomanometer:</u><br>• *# of BPs:* 3<br>• *Interval between measurements:* 2 minutes<br>• *Was average or maximum used for final BP value:* Average<br>• *Rest duration prior to BP measuring:* 5 minutes	Article did not compare between BP groups	QTc Prolonged*	12-lead	Computed (MUSE Cardiology Information System)
**Solanki et al. (2018)[19]**	214	NT: 72 (33.6%)<br>PHT: 0<br>PHIT: 0<br>HT: 142 (66.4%)	For HT participants:<br>Diabetes Mellitus (20%)<br>Hyperlipidemia (5%)<br>Cardiac disease (4%)<br>Smoking (37%)<br>Alcohol Use (22%)<br>Excluded multiple comorbidities<br><br>$\overline{\text{Age}}_p$ = 39.9 years<br>$\widetilde{\text{Age}}_p$ = 7.4 years<br><br>43.5% Female<br><br>No BMIs reported	<u>HT:</u><br>NR<br><br><u>NT:</u><br>SBP < 140 mmHg and DBP < 90 mmHg	<u>Cuffed Sphygmomanometer:</u><br>Details NR	Article did not compare between BP groups	QTc Duration, Lead II*<br>QTc Prolongation, Lead II*<br><br>R wave Amplitude, Lead aVL*<br>S wave Amplitude, Lead V3*	12-lead	Manual (individuals' details NR, NR if blinded to clinical statuses)
**Tosun et al. (2018)[20]**	111	NT: 39 (35.1%)<br>PHT: 0<br>PHIT: 0<br>HT: 72 (64.9%)	Smoking (33.3%)<br>Excluded multiple comorbidities<br><br>$\overline{\text{Age}}_p$ = 52.2 years<br>$\widetilde{\text{Age}}_p$ = 11.2 years<br><br>43.2% Female	<u>HT:</u><br>SBP ≥ 140 mmHg or DBP ≥ 90 mmHg or taking HT medication<br><br><u>NT:</u><br>NR	<u>Cuffed Sphygmomanometer:</u><br>• *# of BPs:* Once in each arm<br>• *Interval between measurements:* NR	Age ($p < 0.05$)<br><br>BMI ($p < 0.05$)	P wave Maximum*<br>P wave Minimum<br>P wave Dispersion*<br>P wave Terminal Force*	12-lead	Method NR

			$\overline{\text{BMI}}_p$ = 27.8 kg/m^2<br>$\widetilde{\text{BMI}}_p$ = 4.5 kg/m^2		• *Was average or maximum used for final BP value:* Maximum<br>• *Rest duration prior to BP measuring:* 5 minutes				
**Eyuboglu et al. (2017)[21]**	548	NT: 159 (29.0%)<br>PHT: 172 (31.4%)<br>PIHT: 0<br>HT: 217 (39.6%)	Coronary Artery Disease (15.7%)<br>Diabetes Mellitus (18.8%)<br>Smoking (20.1%)<br>Excluded multiple cardiovascular diseases<br><br>$\overline{\text{Age}}_p$ = 49.0 years<br>$\widetilde{\text{Age}}_p$ = 5.0 years<br><br>42.0% Female<br><br>No BMIs reported	HT:<br>SBP ≥ 140 mmHg or DBP ≥ 90 mmHg<br><br>PHT:<br>SBP = 120–139 mmHg or DBP = 80–89 mmHg<br><br>NT:<br>SBP < 120 mmHg and DBP < 80 mmHg	Cuffed 24-hour Ambulatory BP:<br>• *Interval between measurements:* 30 minutes<br>• *Was average or maximum used for final BP value:* Average<br>• *Repeated if over 20% of the readings were invalid:* Yes	Age<br>(p < 0.001)	Fragmented QRS*	12-lead	Manual (two cardiologists, blinded to clinical statuses)
**Aeschbacher et al. (2016)[22]**	2,070	NT: 1640 (79.2%)<br>PHT: 0<br>PHIT: 0<br>HT: 430 (20.8%)	Smoking (22.0%)<br>Excluded multiple comorbidities<br><br>$\overline{\text{Age}}_p$ = 36.4 years<br>$\widetilde{\text{Age}}_p$ = NR<br><br>52.9% Female<br><br>$\overline{\text{BMI}}_p$ = 24.6 kg/m^2<br>$\widetilde{\text{BMI}}_p$ = 3.8 kg/m^2	HT:<br>SBP ≥ 140 mmHg or DBP ≥ 90 mmHg or taking HT medication<br><br>NT:<br>NR<br><br>Parameters based on daytime period ambulatory BP readings.	Cuffed 24-hour Ambulatory BP with Oscillometric Device:<br>• *Interval between measurements:* 15 minutes during the day (7:30am to 10pm) and every 30 minutes at night.<br>• *Was average or maximum used for final BP value:* NR<br>• *Repeated if over 20% of the readings were invalid:* Yes	Sex<br>(p < 0.0001)<br><br>Age<br>(p < 0.0001)<br><br>BMI<br>(p < 0.0001)<br><br>Smoking<br>(p = 0.01)	R wave Amplitude, Lead I<br>R wave Amplitude, Lead II<br>S wave Amplitude, Lead aVR*<br>S wave Amplitude, Lead V1*<br>S wave Amplitude, Lead V2*<br><br>T wave Amplitude, Lead V1*	12-lead	Mixed<br><br>Computed (SEMA 200)<br><br>and<br><br>Random manual reviews (one study reviewer, NR if blinded to clinical statuses)

					Participant recorded activities to determine their daytime and nighttime periods.		T wave Amplitude, Lead V2*<br>T wave Amplitude, Lead V4*<br>T wave Amplitude, Lead V5*		
**Avci et al. (2016)**[23]	117	NT: 41 (35.0%)<br>PHT: 0<br>PHIT: 0<br>HT: 76 (65.0%)	Focused on early HT, with HT group only diagnosed for a maximum of 6 months duration.<br>Smoking (35.9%)<br>Excluded multiple comorbidities<br><br>$\overline{\text{Age}}_p$ = 51.6 years<br>$\widetilde{\text{Age}}_p$ = 8.7 years<br><br>46.2% Female<br><br>$\overline{\text{BMI}}_p$ = 29.2 kg/m^2<br>$\widetilde{\text{BMI}}_p$ = 4.0 kg/m^2	<u>HT:</u><br>European Society of Cardiology/ European Society's Grade I and II: SBP 140–179 mmHg and/or DBP 90–109[24]<br><br><u>NT:</u><br>NR	<u>Gold Standard method NR:</u><br>Details NR	No significant differences in reported variables	P wave Maximum	12-lead	Manual (one study investigator, blinded to clinical statuses)
**Pusuroglu et al. (2016)**[25]	184	NT: 43 (23.4%)<br>PHT: 0<br>PHIT: 0<br>HT: 141 (76.6%)	Diabetes Mellitus (17.9%)<br>Smoking (20.8%)<br>Excluded multiple comorbidities<br><br>$\overline{\text{Age}}_p$ = 52.2 years<br>$\widetilde{\text{Age}}_p$ = 10.1 years<br><br>59.2% Female<br><br>$\overline{\text{BMI}}_p$ = 30.1 kg/m^2<br>$\widetilde{\text{BMI}}_p$ = 5.3 kg/m^2	<u>HT:</u><br>Taking HT medication or office BP ≥ 140/90 mmHg, or average daytime 24-hour ambulatory BP ≥ 135/85 mmHg, or average nighttime 24-hour ambulatory BP ≥ 120/70 mmHg.<br><br><u>NT:</u><br>Office BP ≤ 140/90 mmHg, and 24-hour ambulatory BP	<u>Gold Standard office method NR:</u><br>Details NR.<br><br>Plus<br><br><u>Cuffed 24-hour ambulatory BP:</u><br>• *Interval between measurements:* 20 minutes<br>• *Was average or maximum used for final BP value:* Average	BMI (p = 0.005)<br><br>Smoking (p = 0.047)	T wave alternans (+)*	12-lead	Mixed<br><br>Computed (MARS PC Software with MMA algorithm and CardioScan 12.0 DM software)<br><br>And<br><br>Manual (individuals' details NR, NR if blinded

				≤ 130/80 mmHg, and daytime BP ≤ 135/85 and nighttime BP ≤ 120/70.	• *Repeated if over 20% of the readings were invalid:* NR<br><br>Daytime BP was 7 am to 11 pm and nighttime BP was the remaining time.				to clinical statuses)
**Vaidean et al. (2016)[26]**	11,308	NT: 6,918 (61.2%)<br>PHT: 0<br>PIHT: 0<br>HT: 4,390 (38.8%)	Obesity (BMI > 30 kg/m² at 30.6%)<br>Cardiovascular diseases (13.5%)<br>Metabolic Syndrome (38.8%)<br>Smoking (19.6%)<br>Diabetes Mellitus (6.1%)<br>Excluded multiple cardiovascular diseases<br><br>$\overline{Age}$ = 55.6 years<br>$\widetilde{Age}$ = 5.4 years<br><br>67.6% Female<br><br>$\overline{BMI}$ = 28.2 kg/m²<br>$\widetilde{BMI}$ = 4.7 kg/m²	<u>HT:</u><br>SBP ≥ 140 mmHg or DBP ≥ 90 mmHg or taking HT medication or self-reported HT diagnosis.<br><br><u>NT:</u><br>SBP < 140 mmHg and DBP < 90 mmHg	<u>Gold Standard Method NR:</u><br>• *# of BPs:* 2<br>• *Interval between measurements:* NR<br>• *Was average or maximum used for final BP value:* Average<br>• *Rest duration prior to BP measuring:* NR	Article did not compare between BP groups	P wave Duration*<br><br>PR Interval Duration*	12-lead	Mixed<br><br>Computed (AsCARD Mr.Grey v.201 portable Electrocardiograph's software)<br><br>And<br><br>Manual (NR number of study cardiologists, NR if blinded to clinical statuses)
**Ferrucci et al. (2015)[27]**	50	NT: 18 (36.0%)<br>PHT: 0<br>PHIT: 0<br>HT: 32 (64.0%)	No listed comorbidities<br>Excluded multiple comorbidities<br>New HT diagnosis<br>No HT medications<br><br>$\overline{Age}$ = 42.8 years<br>$\widetilde{Age}$ = 9.1 years<br><br>Sex not described<br><br>$\overline{BMI}$ = 26.1 kg/m²<br>$\widetilde{BMI}$ = 4.0 kg/m²	<u>HT:</u><br>Per referenced Europena guidelines:<br>SBP ≥ 140 mmHg and/or DBP ≥ 90 mmHg[28]<br><br><u>NT:</u><br>BP < 140/90 mmHg	<u>Cuffed Automatic Oscillometric Sphygmomanometer:</u><br>• *# of BPs:* 3<br>• *Interval between measurements:* NR<br>• *Was average or maximum used for final BP value:* Average	BMI ($p = 0.043$)	P wave Maximum<br>P wave Minimum<br>P wave Duration<br>P wave Dispersion<br>P wave Area<br>P wave Amplitude, Lead DIII<br><br>PR Interval Duration	12-lead	Manual (two investigators, blinded to clinical statuses)<br><br>Manually used computer Software for ECG

					• *Rest duration prior to BP measuring:*<br>10 minutes		QTc Duration<br>QT Duration<br><br>QRS Duration<br><br>TpTe*<br><br>Ventricular Activation Time		parameter estimations (Adobe Photoshop CS6, with ECGs scanned at 600 dpi)
**Ale et al. (2013)[29]**	252 (120 with ECG data)	NT: 60 (50.0%)<br>PHT: 0<br>PHIT: 0<br>HT: 60 (50.0%)	No listed comorbidities<br>Excluded multiple comorbidities<br><br>$\overline{\text{Age}}_p$ = 53.2 years<br>$\widetilde{\text{Age}}_p$ = 13.3 years<br><br>67.6% Female<br><br>$\overline{\text{BMI}}_p$ = 27.4 kg/m^2<br>$\widetilde{\text{BMI}}_p$ = 5.4 kg/m^2	<u>HT:</u><br>BP ≥ 140/90 mmHg persistently or taking HT medication<br><br><u>NT:</u><br>NR	<u>Gold Standard Method NR:</u><br>Details NR	BMI<br>(p = 0.02)	QTc Duration (Female)*<br>QTc Duration (Male)*<br>QTc Duration (Both)*<br>QTc Prolonged*<br>QT Dispersion*<br>Abnormal QT Dispersion*	12-lead	Manual (individuals' details NR, NR if blinded to clinical statuses)
**Assanelli et al. (2013)[30]**	18,959	NT: 8,658 (45.7%)<br>PHT: 0<br>PHIT: 0<br>HT: 10,301 (54.3%)	Smoking (25.8%)<br>Diabetes Mellitus (8.1%)<br>Hyperlipidemia (30.1%)<br>Excluded multiple cardiovascular diseases<br><br>$\overline{\text{Age}}_p$ = 54.6 years<br>$\widetilde{\text{Age}}_p$ = 11.1 years<br><br>54.8% Female<br><br>$\overline{\text{BMI}}_p$ = 28.1 kg/m^2<br>$\widetilde{\text{BMI}}_p$ = 4.8 kg/m^2	<u>HT:</u><br>SBP ≥ 140 mmHg and/or DBP ≥ 90 mmHg or taking HT medication<br><br><u>NT:</u><br>NR	<u>Cuffed Automatic Sphygmomanometer:</u><br>• *# of BPs:*<br>3<br>• *Interval between measurements:*<br>NR<br>• *Was average or maximum used for final BP value:*<br>Average of last two<br>• *Rest duration prior to BP measuring:*<br>5 minutes	Article did not compare between BP groups	T wave Axis Deviation*	12-lead	Computed (a proprietary measuring tool plus the University of Glasgow 12-Lead ECG diagnostic algorithm)

**Mozos and Filimon (2013)[31]**	60	NT: 53 (88.3%)<br>PHT: 0<br>PHIT: 0<br>HT: 7 (11.7%)	Smoking (51.6%)<br>Excluded multiple comorbidities<br><br>$\overline{Age}$ = 36 years<br>$\widetilde{Age}$ = 10 years<br><br>45.0% Female<br><br>$\overline{BMI}$ = 26 kg/m²<br>$\widetilde{BMI}$ = 4.5 kg/m²	NR	<u>Gold Standard Method NR:</u><br>Details NR<br>.	Article did not compare between BP groups	QTfr Maximum > 50ms*<br><br>T wave Amplitude, Lead V5*<br>T wave Maximum, Lead V5<br>TpTe Maximum, Lead V5*	12-lead	Manual (two observers, blinded to clinical statuses)[32]
**Akintunde et al. (2012)[33]**	210	NT: 70 (33.3%)<br>PHT: 0<br>PHIT: 0<br>HT: 140 (66.7%)	Newly diagnosed with HT<br>Smoking (5.7%)<br>Excluded multiple comorbidities<br><br>$\overline{Age}_p$ = 56.0 years<br>$\widetilde{Age}_p$ = 12.9 years<br><br>52.9% Female<br><br>$\overline{BMI}_p$ = 26.5 kg/m²<br>$\widetilde{BMI}_p$ = 5.5 kg/m²	NR	<u>Gold Standard Method NR:</u><br>Details NR	BMI<br>(p = 0.039)	PR Interval Duration<br><br>QT Maximum*<br>QT Minimum<br>QT Dispersion*<br>QTc Duration<br>QTc Maximum*<br>QTc Dispersion*<br>QTc Prolongation*<br><br>QRS Axis*<br>Mean R wave Amplitude, Lead I*<br>SV1/V2 + RV5/6*	12-lead	Manual (individuals' details NR, NR if blinded to clinical statuses)
**Magnani et al. (2012)[34]**	14,433	NT: 9,565 (66.3%)<br>PHT: 0<br>PHIT: 0<br>HT: 4,868 (33.7%)	Alcohol Use present<br>Smoking (58.3%)<br>Diabetes Mellitus (10.5%)<br>Myocardial Infarction (3.9%)<br>Heart Failure (4.4%)<br>LVH (2.0%)<br>Heart failure and antihypertensive medication taken<br>Excluded cardiac conduction disorders	<u>HT:</u><br>SBP ≥ 140 mmHg or DBP ≥ 90 mmHg or taking HT medication<br><br><u>NT:</u><br>NR	<u>Gold Standard Method NR:</u><br>• *# of BPs:* 3<br>• *Interval between measurements:* NR<br>• *Was average or maximum used for final BP value:* Average of last two	Article did not compare between BP groups	P wave Maximum*<br>P wave Terminal Force, Lead V1*<br><br>PR Interval*	12-lead	Computed (Dalhousie ECG program and GE Marquette 12-SL program's 2001 version)[35]

			$\overline{Age}$ = 54.2 years<br>$\widetilde{Age}$ = 5.8 years<br><br>55.3% Female<br><br>BMI < 25 kg/m² (33.4%)<br>BMI 25 – 30 kg/m² (39.6%)<br>BMI > 30 kg/m² (27.0%)		• *Rest duration prior to BP measuring:* NR				
**Queen et al. (2012)[36]**	1,280	NT: 981 (76.6%)<br>PHT: 0<br>PHIT: 0<br>HT: 299 (23.4%)	Metabolic syndrome (43.4%)<br>Diabetes Mellitus (28.8%)<br>Smoking (27.5%)<br><br>$\overline{Age}$ = 43 years<br>$\widetilde{Age}$ = NR<br><br>67.6% Female<br><br>$\overline{BMI}$ = 30 kg/m²<br>$\widetilde{BMI}$ = NR	<u>HT:</u><br>SBP ≥ 140 mmHg or DBP ≥ 90 mmHg<br><br><u>NT:</u><br>NR	<u>Gold Standard Method NR:</u><br>• *# of BPs:* 3<br>• *Interval between measurements:* 5 minutes<br>• *Was average or maximum used for final BP value:* Average<br>• *Rest duration prior to BP measuring:* NR	Article did not compare between BP groups	QTc Duration*<br><br>Ischemic ECG Abnormalities*	12-lead	Mixed<br><br>Computed (software NR)<br><br>And<br><br>Manual (one cardiologist, NR if blinded to clinical statuses)
**Baumert et al. (2011)[37]**	32	NT: 9 (28.1%)<br>PHT: 0<br>PHIT: 0<br>HT: 23 (71.9%)	No listed comorbidities<br>Excluded multiple comorbidities<br><br>$\overline{Age}_p$ = 42.3 years<br>$\widetilde{Age}_p$ = 12.4 years<br><br>25.0% Female<br><br>$\overline{BMI}_p$ = 26.6 kg/m^2<br>$\widetilde{BMI}_p$ = 5.8 kg/m^2	<u>HT:</u><br>SBP > 140 mmHg or DBP > 90 mmHg<br><br><u>NT:</u><br>SBP < 140 mmHg and DBP < 90 mmHg	<u>Gold Standard Method NR:</u><br>• *# of BPs:* 4 measurements on two occasions<br>• *Interval between measurements:* NR<br>• *Was average or maximum used for final BP value:* Average<br>• *Rest duration prior to BP measuring:* NR	BMI<br>($p < 0.01$)	QT Duration, Lead III<br>QTc Duration, Lead III	Only lead III	Computed (PowerLab and LabChart software)

					Plus<br><br><u>Cuffless Intra-arterial BP measurements with cannula in radial artery:</u> Used to confirm BP measurements. Duration of cannulation NR.				
**Emiroglu et al. (2011)[38]**	160	NT: 80 (50.0%)<br>PHT: 0<br>PHIT: 0<br>HT: 80 (50.0%)	All HT participants with 4 or more years of HT and on HT medication<br>Smoking (25.5%)<br>Excluded multiple comorbidities<br><br>$\overline{Age}_p$ = 51.5 years<br>$\widetilde{Age}_p$ = 12.3 years<br><br>36.9% Female<br><br>$\overline{BMI}_p$ = 23.9 kg/m^2<br>$\widetilde{BMI}_p$ = 4.8 kg/m^2	NR	<u>Gold Standard Method NR:</u><br>Details NR	No significant differences in reported variables	P wave Maximum*<br>P wave Minimum<br>P wave Dispersion*	12-lead	Manual (two investigators, NR if blinded to clinical statuses)
**Ermis et al. (2011)[39]**	129	NT: 46 (35.7%)<br>PHT: 0<br>PHIT: 0<br>HT: 83 (64.3%)	On HT medication<br>Diabetes Mellitus (14.0%)<br>Excluded multiple comorbidities<br><br>$\overline{Age}_p$ = 53.6 years<br>$\widetilde{Age}_p$ = 9.9 years<br><br>54.2% Female<br><br>$\overline{BMI}_p$ = 26.7 kg/m^2<br>$\widetilde{BMI}_p$ = 4.0 kg/m^2	<u>HT:</u><br>SBP ≥ 140 mmHg and/or DBP ≥ 90 mmHg and/or taking HT medication<br><br><u>NT:</u><br>NR	<u>Cuffed Mercury Sphygmomanometer:</u><br>• *# of BPs:* 3<br>• *Interval between measurements:* 1 minute<br>• *Was average or maximum used for final BP value:* Average<br>• *Rest duration prior to BP measuring:* 5 minutes	No significant differences in reported variables	P wave Maximum*<br>P wave Minimum<br>P wave Dispersion*	12-lead	Manual (two observers, blinded to clinical statuses)

**Pshenichnikov et al. (2011)[40]**	302	NT: 176 (58.3%)<br>PHT: 0<br>PHIT: 0<br>HT: 126 (41.7%)	Smoking (20.2%)<br>Dyslipidemia (71.2%)<br>Abdominal Obesity (32.8%)<br>Diabetes Mellitus (7.3%)<br>Microalbuminuria (9.9%)<br>Excluded multiple cardiovascular diseases<br><br>$\overline{Age}$ = 57.4 years<br>$\widetilde{Age}$ = 4.8 years<br><br>100.0% Female<br><br>$\overline{BMI}$ = 27.6 ± 5.0 kg/m²<br>$\widetilde{BMI}$ = 5.0 kg/m^2	<u>HT:</u><br>BP > 140/90 mmHg or taking HT medication or females with diabetes mellitus or microalbuminuria at > 130/85 mmHg<br><br><u>NT:</u><br>NR	<u>Cuffed Mercury Sphygmomanometer:</u><br>• *# of BPs:* 2 on one occasion and 1 more at another occasion<br>• *Interval between measurements:* NR<br>• *Was average or maximum used for final BP value:* Average<br>• *Rest duration prior to BP measuring:* 5 minutes	Article did not compare between BP groups	QTc Duration*<br>QT Dispersion*	12-lead	Manual (two observers, NR if blinded to clinical statuses)
**Anttila et al. (2010)[41]**	5,613	NT: 3,938 (70.2%)<br>PHT: 0<br>PHIT: 0<br>HT: 1,675 (29.8%)	Smoking (21.6%)<br>Chronic Obstructive Pulmonary Disease (1.3%)<br>Diabetes Mellitus (5.3%)<br>Left or Right Ventricular Hypertrophy (14.6%)<br>Coronary Heart Disease (8.0%)<br>Myocardial Infarction (2.3%)<br>Anti-HT medication taken<br>Excluded cardiac conduction disorders<br><br>$\overline{Age}_p$ = 51.9 years<br>$\widetilde{Age}_p$ = 14.3 years<br><br>56.1% Female<br><br>$\overline{BMI}_p$ = 27.0 kg/m^2<br>$\widetilde{BMI}_p$ = 4.6 kg/m^2	<u>HT:</u><br>BP ≥ 140/90 mmHg<br><br><u>NT:</u><br>NR	<u>Cuffed Mercury Sphygmomanometer:</u><br>• *# of BPs:* 2<br>• *Interval between measurements:* 2 minutes<br>• *Was average or maximum used for final BP value:* Average<br>• *Rest duration prior to BP measuring:* 5 minutes	Article did not compare between BP groups	Poor R wave Progression, Leads V2, V3* for female (NS for male)	12-lead	Mixed<br><br>Computed (Magellan software program)<br><br>And<br><br>Manual (individuals' details NR, NR if blinded to clinical statuses)

**Sriratana viriyakul (2010)[42]**	1,485	NT: 1,316 (88.6%)<br>PHT: 0<br>PHIT: 0<br>HT: 169 (11.4%)	No exclusions listed<br>Diabetes (1.7%)<br>Impaired fasting glucose (14.6%)<br>Hypercholesterolemia (56.4%)<br>Smoking (12.7%)<br>Obesity (19.4%)<br><br>$\overline{Age}$ = 34.4 years<br>$\widetilde{Age}$ = 5.4 years<br><br>57.0% Female<br><br>$\overline{BMI}_p = 22.2$ kg/m^2<br>$\widetilde{BMI}_p = 3.5$ kg/m^2	HT:<br>SBP ≥ 140 mmHg and/or DBP ≥ 90 mmHg or taking HT medication in last week.<br><br>NT:<br>NR	Cuffed Mercury Sphygmomanometer:<br>• *# of BPs:* 2 or 3 if the first two were over 10 mmHg apart<br>• *Interval between measurements:* NR<br>• *Was average or maximum used for final BP value:* Average<br>• *Rest duration prior to BP measuring:* 5 minutes	Article did not compare between BP groups	Q/QS wave Abnormality<br><br>T wave Inversion<br><br>ST Depression<br><br>AV Conduction Defect:<br>Third Degree Block<br>Second Degree Block<br>First Degree Block<br><br>Ventricular conduction defect:<br>Left Bundle Branch Block<br>Right Bundle Branch Block*	12-lead	Manual (one general practitioner and one cardiologist, NR if blinded to clinical statuses)

APPENDIX 4

Seven summary tables of results from the 35 included articles grouped by types of ECG features:

Table A. Summary of papers that evaluated P wave features. NT = normotensive, PHT = prehypertensive, HT = hypertensive, PIHT = pregnancy-induced hypertension, NS = not significant, BP = blood pressure. $\bar{x}$ = mean of variable x reported by the article, where x can be BMI or age. $\tilde{x}$ = standard deviation reported by the article, where x can be BMI or age. $\bar{x}_{\text{p}}$= calculated pooled mean of variable x, where x can be BMI or age. $\tilde{x}_{\text{p}}$= calculated pooled standard deviation of variable x, where x can be BMI or age. NR = Not reported.

**Study**	**Sample Size**	**Age & Sex**	**# of Comorbidities/ Health Considerations**	**NT/PHT/PIHT /HT**	**Leads**	**Sampling Frequency**	**Position During Collection**	**P wave Features, Significance with BP**
Hassing et al. (2019)	1,449	$\overline{\text{Age}}$ = 22.7 years<br>$\widetilde{\text{Age}}$ = 3.0 years<br><br>26.3% Female	After medical screening, only healthy included.<br><br>$\overline{\text{BMI}}_{\text{p}}$ for SBP: 22.7 kg/m^2<br>$\widetilde{\text{BMI}}_{\text{p}}$ for SBP: 2.5 kg/m^2	NT: 1,197 (82.6%)<br>PHT: 252 (17.4%)<br>(by systolic BP)<br>PIHT: 0<br>HT: 0	12-lead<br><br>V1	NR	BP: Supine<br>ECG: Supine	Maximum in the 12 leads, NS<br>Dispersion in the 12 leads, NS<br>Area in Lead V1, ↑ (***p* < 0.001**)
Sun et al. (2019)	11,264	$\overline{\text{Age}}_{\text{p}}$ = 53.7 years<br>$\widetilde{\text{Age}}_{\text{p}}$ = 10.6 years<br><br>54.5% Female	Diabetes Mellitus (10.3%)<br>Smoke (35.2%)<br>Alcohol use (22.3%)<br>Anti-HT medication (16.2%)<br>Anti-arrhythmia medication (0.6%)<br>Echocardiogram abnormalities<br>History of MI (1.1%)<br>History of HF (0.8%)<br>Mitral stenosis/regurgitation (1.6%)<br>Excluded multiple cardiovascular diseases<br><br>$\overline{\text{BMI}}_{\text{p}}$ = 24.8 kg/m^2<br>$\widetilde{\text{BMI}}_{\text{p}}$ = 3.7 kg/m^2	NT: 3,111 (27.6%)<br>PHT: 2,406 (21.4%)<br>PIHT: 0<br>HT: 5,747 (51.0%)	12-lead	NR	BP: Sitting<br>ECG: NR	Prolongation in the 12 leads, ↑ (***p* < 0.001**)
Tosun et al. (2018)	111	$\overline{\text{Age}}_{\text{p}}$ = 52.2 years<br>$\widetilde{\text{Age}}_{\text{p}}$ = 11.2 years	Smoke (33.3%)<br>Multiple comorbidity exclusions	NT: 39 (35.1%)<br>PHT: 0<br>PHIT: 0<br>HT: 72 (64.9%)	12-lead	NR	BP: Sitting<br>ECG: NR	Maximum in the 12 leads, ↑ (***p* < 0.05**)<br>Minimum in the 12 leads, NS<br>Dispersion in the 12 leads, ↑ (***p* < 0.05**)

		43.2% Female	$\overline{\text{BMI}}_p$ = 27.8 kg/m^2<br>$\widetilde{\text{BMI}}_p$ = 4.5 kg/m^2					Terminal Force in the 12 leads, ↑ (**p < 0.05**)
Avci et al. (2016)	117	$\overline{\text{Age}}_p$ = 51.6 years<br>$\widetilde{\text{Age}}_p$ = 8.7 years<br><br>46.2% Female	Focused on early HT, with HT group only diagnosed for a maximum of 6 months duration.<br>Smoke (35.9%)<br>Excluded multiple comorbidities<br><br>$\overline{\text{BMI}}_p$ = 29.2 kg/m^2<br>$\widetilde{\text{BMI}}_p$ = 4.0 kg/m^{22}	NT: 41 (35.0%)<br>PHT: 0<br>PHIT: 0<br>HT: 76 (65.0%)	12-lead	NR	BP: NR<br>ECG: Supine	Maximum in the 12 leads, NS
Gazi et al. (2016)	41	$\overline{\text{Age}}_p$ = 28.2 years<br>$\widetilde{\text{Age}}_p$ = 5.7 years<br><br>100.0% Female	No listed comorbidities<br>Multiple comorbidity exclusions<br>Pregnancy<br><br>No BMIs reported	NT: 24 (58.5%)<br>PHT: 0<br>PIHT: 17 (41.5%)<br>HT: 0	12-lead	NR	BP: NR<br>ECG: NR	Maximum in the 12 leads, NS<br>Minimum in the 12 leads, NS<br>Dispersion in the 12 leads, NS
Vaidean et al. (2016)	11,308	$\overline{\text{Age}}$ = 55.6 years<br>$\widetilde{\text{Age}}$ = 5.4 years<br><br>67.6% Female	Obesity (BMI > 30 kg/m^2 at 30.6%)<br>Cardiovascular diseases (13.5%)<br>Metabolic Syndrome (38.8%)<br>Smoke (19.6%)<br>Diabetes Mellitus (6.1%)<br>Excluded multiple cardiovascular diseases<br><br>$\overline{\text{BMI}}$ = 28.2 kg/m^2<br>$\widetilde{\text{BMI}}$ = 4.7 kg/m^2	NT: 6,918 (61.2%)<br>PHT: 0<br>PIHT: 0<br>HT: 4,390 (38.8%)	12-lead	NR	BP: Sitting<br>ECG: Supine	Mean Duration in the 12 leads, ↑ (**p < 0.05**)
Ferrucci et al. (2015)	50	$\overline{\text{Age}}$ = 42.8 years<br>$\widetilde{\text{Age}}$ = 9.1 years<br><br>Sex not described	No listed comorbidities<br>Multiple comorbidity exclusions<br>New HT diagnosis<br>No HT medications	NT: 18 (36.0%)<br>PHT: 0<br>PHIT: 0<br>HT: 32 (64.0%)	12-lead	NR	BP: Sitting<br>ECG: Supine	Maximum in the 12 leads, NS<br>Minimum in the 12 leads, NS<br>Average Duration & Duration in each of the 12 leads NS<br>Dispersion in the 12 leads, NS

			$\overline{\text{BMI}}$ = 26.1 kg/m^2<br>$\widetilde{\text{BMI}}$ = 4.0 kg/m^2					Area in the 12 leads, NS<br>Amplitude in Lead III NS
Chávez et al. (2014)	656	Children 8 – 11 years (Tanner stage 1)<br><br>50.3% Female	Excluded multiple comorbidities<br><br>Obese (16.8%)<br>No BMIs reported	NT: 394 (60.0%)<br>PHT: 199 (30.3%)<br>PIHT: 0<br>HT: 63 (9.7%)	12-lead	NR	BP: NR<br>ECG: NR	Dispersion in the 12 leads, ↑ (***p* < 0.001**) for HT vs. NT<br>Dispersion in the 12 leads, ↑ (***p* = 0.001**) for HT vs. PHT<br>Dispersion in the 12 leads, ↑ (***p* = 0.011**) for PHT vs. NT
Kirbas et al. (2014)	88	$\overline{\text{Age}}_p$ = 28.0 years<br>$\widetilde{\text{Age}}_p$ = 5.3 years<br><br>100.0% Female	Pregnancy<br>Preeclampsia<br>Excluded multiple comorbidities<br><br>$\overline{\text{BMI}}_p$ = 29.3 kg/m^2<br>$\widetilde{\text{BMI}}_p$ = 2.8 kg/m^2	NT: 30 (34.1%)<br>PHT: 0<br>PIHT: 58 (65.9%)<br>HT: 0	12-lead	NR	BP: NR<br>ECG: NR	(*For between all three groups of NT vs. mild vs. severe preeclampsia*)<br><br>Maximum in the 12 leads, NS<br>Minimum in the 12 leads, ↓ (***p* < 0.001**)<br>Dispersion in the 12 leads, ↑ (***p* < 0.001**)
Chávez et al. (2013)	515	Children 8 – 11 years (Tanner stage 1)<br><br>No Sex percentages	No listed comorbidities<br>Excluded multiple comorbidities<br><br>No BMIs reported	NT: 333 (64.7%)<br>PHT: 156 (30.3%)<br>PIHT: 0<br>HT: 26 (5.0%)	12-lead	NR	BP: Sitting<br>ECG: NR	Dispersion in the 12 leads, ↑ (***p* < 0.001**) for HT vs. NT<br>Dispersion in the 12 leads, NS for HT vs. PHT<br>Dispersion in the 12 leads, ↑ (***p* < 0.001**) for PHT vs. NT
Magnani et al. (2012)	14,433	$\overline{\text{Age}}$ = 54.2 years<br>$\widetilde{\text{Age}}$ = 5.8 years<br><br>55.3% Female	Alcohol Use present<br>Smoke (58.3%)<br>Diabetes Mellitus (10.5%)<br>Myocardial Infarction (3.9%)<br>Heart Failure (4.4%)<br>LVH (2.0%)<br>Heart failure and antihypertensive medication taken<br>Excluded cardiac conduction disorders<br><br>BMI < 25 kg/m² (33.4%)	NT: 9,565 (66.3%)<br>PHT: 0<br>PHIT: 0<br>HT: 4,868 (33.7%)	12-lead<br><br>V1	NR	BP: Sitting<br>ECG: NR	Maximum in the 12 leads, ↑ (***p* < 0.001**)<br>Terminal Force in Lead V1, ↑ (***p* < 0.001**)

			BMI 25 – 30 kg/m² (39.6%)<br>BMI > 30 kg/m² (27.0%)					
Yildirim et al. (2012)	48	$\overline{\text{Age}}_p$ = 44.4 years<br>$\widetilde{\text{Age}}_p$ = 9.7 years<br><br>68.8% Female	Excluded multiple comorbidities<br><br>$\overline{\text{BMI}}_p$ = 30.0 kg/m^2<br>$\widetilde{\text{BMI}}_p$ = 3.0 kg/m^2	NT: 24 (50.0%)<br>PHT: 0<br>PHIT: 0<br>HT: 24 (50.0%)	12-lead	NR	BP: Sitting<br>ECG: Supine	Maximum in the 12 leads, ↑ (***p* < 0.05**)<br>Minimum in the 12 leads, NS<br>Dispersion in the 12 leads, ↑ (***p* < 0.05**)
Emiroglu et al. (2011)	160	$\overline{\text{Age}}_p$ = 51.5 years<br>$\widetilde{\text{Age}}_p$ = 12.3 years<br><br>36.9% Female	All HT participants with 4 or more years of HT and on HT medication<br>Smoke (25.5%)<br>Excluded multiple comorbidities<br><br>$\overline{\text{BMI}}_p$ = 23.9 kg/m^2<br>$\widetilde{\text{BMI}}_p$ = 4.8 kg/m^2	NT: 80 (50.0%)<br>PHT: 0<br>PHIT: 0<br>HT: 80 (50.0%)	12-lead	1000 Hz	BP: NR<br>ECG: NR	Maximum in the 12 leads, ↑ (***p* = 0.01**)<br>Minimum in the 12 leads, NS<br>Dispersion in the 12 leads, ↑ (***p* < 0.001**)
Ermis et al. (2011)	129	$\overline{\text{Age}}_p$ = 53.6 years<br>$\widetilde{\text{Age}}_p$ = 9.9 years<br><br>54.2% Female	On HT medication<br>Diabetes Mellitus (14.0%)<br>Excluded multiple comorbidities<br><br>$\overline{\text{BMI}}_p$ = 26.7 kg/m^2<br>$\widetilde{\text{BMI}}_p$ = 4.0 kg/m^2	NT: 46 (35.7%)<br>PHT: 0<br>PHIT: 0<br>HT: 83 (64.3%)	12-lead	NR	BP: NR<br>ECG: Supine	Maximum in the 12 leads, ↑ (***p* < 0.001**)<br>Minimum in the 12 leads, NS<br>Dispersion in the 12 leads, ↑ (***p* < 0.001**)

0
1

Table B. Summary of papers that evaluated PR interval features. NT = normotensive, PHT = prehypertensive, HT = hypertensive, PIHT = pregnancy-induced hypertension, NS = not significant, BP = blood pressure. $\bar{x}$ = mean of variable x reported by the article, where x can be BMI or age. $\tilde{x}$ = standard deviation reported by the article, where x can be BMI or age. $\bar{x}_p$= calculated pooled mean of variable x, where x can be BMI or age. $\tilde{x}_p$= calculated pooled standard deviation of variable x, where x can be BMI or age. NR = Not reported.

**Study**	**Sample Size**	**Age & Sex**	**# of Comorbidities**	**NT/PHT/HT**	**Leads**	**Sampling Frequency**	**Position During Collection**	**PR Interval Features, Significance with BP**
Hassing et al. (2019)	1,449	$\overline{Age}$ = 22.7 years<br>$\widetilde{Age}$ = 3.0 years<br><br>26.3% Female	After medical screening, only healthy included.<br><br>$\overline{BMI}_p$ for SBP: 22.7 kg/m^2<br>$\widetilde{BMI}_p$ for SBP: 2.5 kg/m^2	NT: 1,197 (82.6%)<br>PHT: 252 (17.4%)<br>(by systolic BP)<br>PIHT: 0<br>HT: 0	12-lead	NR	BP: Supine<br>ECG: Supine	Duration in the 12 leads, NS
Vaidean et al. (2016)	11,308	$\overline{Age}$ = 55.6 years<br>$\widetilde{Age}$ = 5.4 years<br><br>67.6% Female	Obesity (BMI > 30 kg/m² at 30.6%)<br>Cardiovascular diseases (13.5%)<br>Metabolic Syndrome (38.8%)<br>Smoke (19.6%)<br>Diabetes Mellitus (6.1%)<br>Excluded multiple cardiovascular diseases<br><br>$\overline{BMI}$ = 28.2 kg/m²<br>$\widetilde{BMI}$ = 4.7 kg/m^2	NT: 6,918 (61.2%)<br>PHT: 0<br>PIHT: 0<br>HT: 4,390 (38.8%)	12-lead	NR	BP: Sitting<br>ECG: Supine	Duration in the 12 leads, ↑ (***p* < 0.0001**)
Ferrucci et al. (2015)	50	$\overline{Age}$ = 42.8 years<br>$\widetilde{Age}$ = 9.1 years<br><br>Sex not described	No listed comorbidities<br>Multiple comorbidity exclusions<br>New HT diagnosis<br>No HT medications<br><br>$\overline{BMI}$ = 26.1 kg/m^2<br>$\widetilde{BMI}$ = 4.0 kg/m^2	NT: 18 (36.0%)<br>PHT: 0<br>PHIT: 0<br>HT: 32 (64.0%)	12-lead	NR	BP: Sitting<br>ECG: Supine	Duration in the 12 leads, NS
Akintunde et al. (2012)	210	$\overline{Age}_p$ = 56.0 years<br>$\widetilde{Age}_p$ = 12.9 years<br><br>52.9% Female	Newly diagnosed with HT<br>Smoke (5.7%)<br>Excluded multiple comorbidities<br><br>$\overline{BMI}_p$ = 26.5 kg/m^2	NT: 70 (33.3%)<br>PHT: 0<br>PHIT: 0<br>HT: 140 (66.7%)	12-lead	NR	BP: NR<br>ECG: NR	Duration in the 12 leads, NS

			$\widetilde{BMI}_p$ = 5.5 kg/m^2					
Anigbogu et al. (2012)	78	No mean age reported<br><br>Sex not described	Excluded any disease conditions aside from hypertension.<br>Excluded significant alcohol use, smoking, and medications which affected cardiovascular function.<br><br>No BMIs reported	NT: 39 (50.0%)<br>PHT: 0<br>PHIT: 0<br>HT: 39 (50.0%)	12-lead	NR	BP: Sitting<br>ECG: NR	Duration in the 12 leads, NS
Magnani et al. (2012)	14,433	$\overline{Age}$ = 54.2 years<br>$\widetilde{Age}$ = 5.8 years<br><br>55.3% Female	Alcohol Use present<br>Smoke (58.3%)<br>Diabetes Mellitus (10.5%)<br>Myocardial Infarction (3.9%)<br>Heart Failure (4.4%)<br>LVH (2.0%)<br>Heart failure and antihypertensive medication taken<br>Excluded cardiac conduction disorders<br><br>BMI < 25 kg/m^2 (33.4%)<br>BMI 25 – 30 kg/m^2 (39.6%)<br>BMI > 30 kg/m^2 (27.0%)	NT: 9,565 (66.3%)<br>PHT: 0<br>PHIT: 0<br>HT: 4,868 (33.7%)	12-lead	NR	BP: Sitting<br>ECG: NR	Duration in the 12 leads, ↑ (**p < 0.001**)

Table C. Summary of papers that evaluated QT interval features. NT = normotensive, PHT = prehypertensive, HT = hypertensive, PIHT = pregnancy-induced hypertension, NS = not significant, BP = blood pressure. $\bar{x}$ = mean of variable x reported by the article, where x can be BMI or age. $\tilde{x}$ = standard deviation reported by the article, where x can be BMI or age. $\bar{x}_p$ = calculated pooled mean of variable x, where x can be BMI or age. $\tilde{x}_p$ = calculated pooled standard deviation of variable x, where x can be BMI or age. NR = Not reported.

Study	Sample Size	Age & Sex	# of Comorbidities	NT/PHT/HT	Leads	Sampling Frequency	Position During Collection	QT Interval Features, Significance with BP
Hassing et al. (2019)	1,449	$\overline{Age}$ = 22.7 years<br>$\widetilde{Age}$ = 3.0 years<br><br>26.3% Female	After medical screening, only healthy included.<br><br>$\overline{BMI}_p$ for SBP: 22.7 kg/m^2<br>$\widetilde{BMI}_p$ for SBP: 2.5 kg/m^2	NT: 1,197 (82.6%)<br>PHT: 252 (17.4%)<br>(by systolic BP)<br>PIHT: 0<br>HT: 0	12-lead	NR	BP: Supine<br>ECG: Supine	QTcF Duration in the 12 leads, ↓ (***p* = 0.005**)
Sun et al. (2019)	10,553	$\overline{Age}_p$ = 53.7 years<br>$\widetilde{Age}_p$ = 10.4 years<br><br>54.8% Female	Smoke (35.2%)<br>Alcohol Use (22.2%)<br>Heart Disease History (9.8%)<br>Take any medication (53.2%)<br>Multiple comorbidity exclusions<br><br>$\overline{BMI}_p$ = 24.8 kg/m^2<br>$\widetilde{BMI}_p$ = 3.6 kg/m^2	NT: 5192 (49.2%)<br>PHT: 0<br>PHIT: 0<br>HT: 5,341 (50.8%)	12-lead	NR	BP: NR<br>ECG: NR	QTc Prolonged in the 12 leads, ↑ (***p* < 0.001**)
Solanki et al. (2018)	214	$\overline{Age}_p$ = 39.9 years<br>$\widetilde{Age}_p$ = 7.4 years<br><br>43.5% Female	Diabetes Mellitus (20%)<br>Hyperlipidemia (5%)<br>Cardiac disease (4%)<br>Smoke (37%)<br>Alcohol Use (22%)<br>Multiple comorbidity exclusions<br><br>No BMIs reported	NT: 72 (33.6%)<br>PHT: 0<br>PHIT: 0<br>HT: 142 (66.4%)	12-lead<br><br>Lead II	NR	BP: NR<br>ECG: Supine	QTc Duration in Lead II, ↑ (***p* < 0.001**)<br>QTc Prolonged in Lead II, ↑ (***p* < 0.001**)

Gazi et al. (2016)	41	$\overline{\text{Age}}_p$ = 28.2 years<br>$\widetilde{\text{Age}}_p$ = 5.7 years<br><br>100.0% Female	No listed comorbidities<br>Multiple comorbidity exclusions<br>Pregnancy<br><br>No BMIs reported	NT: 24 (58.5%)<br>PHT: 0<br>PIHT: 17 (41.5%)<br>HT: 0	12-lead	NR	BP: NR<br>ECG: NR	QTc Maximum in the 12 leads, NS<br>QTc Minimum in the 12 leads, NS<br>QT Maximum in the 12 leads, ↑ (***p* = 0.03**)<br>QT Minimum in the 12 leads, NS<br>QT Dispersion in the 12 leads, NS
Kirbas et al. (2016)	96	$\overline{\text{Age}}_p$ = 27.6 years<br>$\widetilde{\text{Age}}_p$ = 5.1 years<br><br>100.0% Female	Pregnancy<br>Preeclampsia<br>Excluded multiple comorbidities<br><br>$\overline{\text{BMI}}_p$ = 29.3 kg/m^2<br>$\widetilde{\text{BMI}}_p$ = 3.5 kg/m^2	NT: 32 (33.3%)<br>PHT: 0<br>PIHT: 64 (66.6%)<br>HT: 0	12-lead	NR	BP: NR<br>ECG: NR	*(NT vs. mild preeclampsia;<br>NT vs. severe preeclampsia)*<br><br>QTc Maximum in the 12 leads, ↑ (NS; ***p* < 0.001**)<br>QTc Minimum in the 12 leads, (NS; NS)<br>QTc Dispersion in the 12 leads, ↑ (***p* = 0.044; *p* < 0.001**)<br>QTc Mean Duration in the 12 leads, ↑ (NS, ***p* = 0.007**)
Ferrucci et al. (2015)	50	$\overline{\text{Age}}$ = 42.8 years<br>$\widetilde{\text{Age}}$ = 9.1 years<br><br>Sex not described	No listed comorbidities<br>Multiple comorbidity exclusions<br>New HT diagnosis<br>No HT medications<br><br>$\overline{\text{BMI}}$ = 26.1 kg/m^2<br>$\widetilde{\text{BMI}}$ = 4.0 kg/m^2	NT: 18 (36.0%)<br>PHT: 0<br>PHIT: 0<br>HT: 32 (64.0%)	12-lead	NR	BP: Sitting<br>ECG: Supine	QTc Duration in the 12 leads, NS<br>QT Duration in the 12 leads, NS
Tanindi et al. (2015)	84	$\overline{\text{Age}}_p$ = 51.5 years<br>$\widetilde{\text{Age}}_p$ = 13.1 years<br><br>56.0% Female	No listed comorbidities<br>Multiple comorbidity exclusions<br><br>$\overline{\text{BMI}}_p$ = 26.3 kg/m^2<br>$\widetilde{\text{BMI}}_p$ = 2.9 kg/m^2	NT: 37 (44.0%)<br>PHT: 47 (56.0%)<br>PIHT: 0<br>HT: 0	12-lead	NR	BP: Sitting<br>ECG: Supine	QTc Maximum in the 12 leads, NS<br>QTc Minimum in the 12 leads, NS<br>QTc Dispersion in the 12 leads, ↑ (***p* < 0.001**)

Ale et al. (2013)	252 (120 with ECG data)	$\overline{Age}_p$ = 53.2 years<br>$\widetilde{Age}_p$ = 13.3 years<br><br>67.6% Female	No listed comorbidities<br>Multiple comorbidity exclusions<br>$\overline{BMI}_p$ = 27.4 kg/m^2<br>$\widetilde{BMI}_p$ = 5.4 kg/m^2	NT: 60 (50.0%)<br>PHT: 0<br>PHIT: 0<br>HT: 60 (50.0%)	12-lead	NR	BP: NR<br>ECG: NR	QTc Duration (female) in the 12 leads, ↑ (***p* = 0.004**)<br>QTc Duration (male) in the 12 leads, ↑ (***p* < 0.00**)<br>QTc Duration (both) in the 12 leads, ↑ (***p* < 0.00**)<br>QTc Prolonged in the 12 leads, ↑% (***p* < 0.0001**)<br>QT Dispersion in the 12 leads, ↑ (***p* < 0.0001**)<br>Abnormal QT Dispersion in the 12 leads, ↑% (***p* < 0.02**)
Mozos and Filimon (2013)	60	$\overline{Age}$ = 36 years<br>$\widetilde{Age}$ = 10 years<br><br>45.0% Female	Smoke (51.6%)<br>Excluded multiple comorbidities<br><br>$\overline{BMI}$ = 26 kg/m^2<br>$\widetilde{BMI}$ = 4.5 kg/m^2	NT: 53 (88.3%)<br>PHT: 0<br>PHIT: 0<br>HT: 7 (11.7%)	12-lead	NR	BP: NR<br>ECG: NR	QTfr Maximum > 50ms in the 12 leads, (***p* = 0.012**)
Akintunde et al. (2012)	210	$\overline{Age}_p$ = 56.0 years<br>$\widetilde{Age}_p$ = 12.9 years<br><br>52.9% Female	Newly diagnosed with HT<br>Smoke (5.7%)<br>Excluded multiple comorbidities<br><br>$\overline{BMI}_p$ = 26.5 kg/m^2<br>$\widetilde{BMI}_p$ = 5.5 kg/m^2	NT: 70 (33.3%)<br>PHT: 0<br>PHIT: 0<br>HT: 140 (66.7%)	12-lead	NR	BP: NR<br>ECG: NR	QTc Maximum in the 12 leads, ↑ (***p* = 0.019**)<br>QTc Dispersion in the 12 leads, ↑ (***p* < 0.001**)<br>QTc Duration in the 12 leads, NS<br>QTc Prolongation in the 12 leads, ↑ (***p* = 0.01**)<br>QT Maximum in the 12 leads, ↑ (***p* = 0.046**)<br>QT Minimum in the 12 leads, NS<br>QT Dispersion in the 12 leads, ↑ (***p* = 0.002**)
Anigbogu et al. (2012)	78	No mean age reported<br><br>Sex not described	Excluded any disease conditions aside from hypertension.<br>Excluded significant alcohol use, smoking, and medications which affected	NT: 39 (50.0%)<br>PHT: 0<br>PHIT: 0<br>HT: 39 (50.0%)	12-lead	NR	BP: Sitting<br>ECG: NR	QT Duration in the 12 leads, NS

			cardiovascular function.<br><br>No BMIs reported					
Queen et al. (2012)	1,280	$\overline{Age}$ = 43 years<br>$\widetilde{Age}$ = NR<br><br>67.6% Female	Metabolic syndrome (43.4%)<br>Diabetes Mellitus (28.8%)<br>Smoke (27.5%)<br><br>$\overline{BMI}$ = 30 kg/m²<br>$\widetilde{BMI}$ = NR	NT: 981 (76.6%)<br>PHT: 0<br>PHIT: 0<br>HT: 299 (23.4%)	12-lead	NR	BP: NR<br>ECG: Supine	QTc Duration in the 12 leads, ↑ (***p* < 0.001**)
Baumert et al. (2011)	32	$\overline{Age}_p$ = 42.3 years<br>$\widetilde{Age}_p$ = 12.4 years<br><br>25.0% Female	No listed comorbidities<br>Multiple comorbidity exclusions<br><br>$\overline{BMI}_p$ = 26.6 kg/m^2<br>$\widetilde{BMI}_p$ = 5.8 kg/m^2	NT: 9 (28.1%)<br>PHT: 0<br>PHIT: 0<br>HT: 23 (71.9%)	Body surface ECG (Lead III)	1000 Hz	BP: NR<br>ECG: NR	QTc Duration in Lead III, NS<br>QT Duration in Lead III, NS
Pshenichnikov et al. (2011)	302	$\overline{Age}$ = 57.4 years<br>$\widetilde{Age}$ = 4.8 years<br><br>100.0% Female	Smoke (20.2%)<br>Dyslipidemia (71.2%)<br>Abdominal Obesity (32.8%)<br>Diabetes Mellitus (7.3%)<br>Microalbuminuria (9.9%)<br>Excluded multiple cardiovascular diseases<br><br>$\overline{BMI}$ = 27.6 ± 5.0 kg/m²<br>$\widetilde{BMI}$ = 5.0 kg/m^2	NT: 176 (58.3%)<br>PHT: 0<br>PHIT: 0<br>HT: 126 (41.7%)	12-lead	NR	BP: NR<br>ECG: NR	QTc Duration in the 12 leads, ↑ (***p* < 0.001**)<br><br>QT Dispersion in the 12 leads, ↑ (***p* < 0.001**)

Zhao et al. (2010)	121<br><br>(83 without LVH)	$\overline{\text{Age}}_p$ = 57.3 years<br>$\widetilde{\text{Age}}_p$ = 12.0 years<br><br>44.6% Female	No listed comorbidities<br>Multiple comorbidity exclusions<br><br>No BMIs reported	NT: 42 (50.6%)<br>PHT: 0<br>PHIT: 0<br>HT: 41 (49.4%)	12-lead<br><br>V4-V6	NR	BP: NR<br>ECG: NR	QTc Peak in Leads V4 – V6,NS<br>QTc Duration in Leads V4 – V6,NS

Table D. Summary of papers that evaluated QRS features. LVH = left ventricular hypertrophy, CAD = coronary artery disease, NT = normotensive, PHT = prehypertensive, HT = hypertensive, PIHT = pregnancy-induced hypertension, NS = not significant, BP = blood pressure. $\bar{x}$ = mean of variable x reported by the article, where x can be BMI or age. $\tilde{x}$ = standard deviation reported by the article, where x can be BMI or age. $\bar{x}_p$ = calculated pooled mean of variable x, where x can be BMI or age. $\tilde{x}_p$ = calculated pooled standard deviation of variable x, where x can be BMI or age. NR = Not reported.

**Study**	**Sample Size**	**Age & Sex**	**# of Comorbidities**	**NT/PHT/HT**	**Leads**	**Sampling Frequency**	**Position During Collection**	**QRS Features, Significance with BP**
Dzikowicz & Carey (2019)	77	$\overline{\text{Age}}$ = 43.4 years<br>$\widetilde{\text{Age}}$ = 7.8 years<br><br>3.9% Female	Sleep Apnea (4%)<br>Left Ventricular Hypertrophy (3%)<br>Smoke (13%)<br><br>$\overline{\text{BMI}}$ = 29.4 kg/m²<br>$\widetilde{\text{BMI}}$ = 4.4 kg/m²	NT: 23 (29.9%)<br>PHT: 29 (37.7%)<br>PIHT: 0<br>HT: 25 (32.4%)	12-lead	60 Hz	BP: Sitting<br>ECG: 24-hour holter	QRS Duration in the 12 leads, NS
Hassing et al. (2019)	1,449	$\overline{\text{Age}}$ = 22.7 years<br>$\widetilde{\text{Age}}$ = 3.0 years<br><br>26.3% Female	After medical screening, only healthy included.<br><br>$\overline{\text{BMI}}_p$ for SBP: 22.7 kg/m²<br>$\widetilde{\text{BMI}}_p$ for SBP: 2.5 kg/m²	NT: 1,197 (82.6%)<br>PHT: 252 (17.4%)<br>(by systolic BP)<br>PIHT: 0<br>HT: 0	12-lead	NR	BP: Supine<br>ECG: Supine	QRS Duration in the 12 leads, ↑ (***p* < 0.001**)<br>R wave Axis in the 12 leads, NS
Ferrucci et al. (2015)	50	$\overline{\text{Age}}$ = 42.8 years<br>$\widetilde{\text{Age}}$ = 9.1 years<br><br>Sex not described	No listed comorbidities<br>Multiple comorbidity exclusions<br>New HT diagnosis<br>No HT medications<br><br>$\overline{\text{BMI}}$ = 26.1 kg/m²<br>$\widetilde{\text{BMI}}$ = 4.0 kg/m²	NT: 18 (36.0%)<br>PHT: 0<br>PHIT: 0<br>HT: 32 (64.0%)	12-lead	NR	BP: Sitting<br>ECG: Supine	QRS Duration in the 12 leads, NS
Anigbogu et al. (2012)	78	No mean age reported<br><br>Sex not described	Excluded any disease conditions aside from hypertension.<br>Excluded significant alcohol use, smoking, and medications	NT: 39 (50.0%)<br>PHT: 0<br>PHIT: 0<br>HT: 39 (50.0%)	12-lead	NR	BP: Sitting<br>ECG: NR	QRS Duration in the 12 leads, ↑ (***p* < 0.01**)

			which affected cardiovascular function.<br><br>No BMIs reported					
Bekar et al. (2019)	353	$\overline{\text{Age}}_p$ = 51.6 years<br>$\widetilde{\text{Age}}_p$ = 7.3 years<br><br>63.7% Female	Hyperlipidemia (35.9%)<br>Smoke (14.6%)<br>Multiple comorbidity exclusions<br><br>$\overline{\text{BMI}}_p$ = 30.9 kg/m^2<br>$\widetilde{\text{BMI}}_p$ = 5.1 kg/m^2	NT: 153 (43.3%)<br>PHT: 0<br>PHIT: 0<br>HT: 200 (56.7%)	12-lead	150 Hz	BP: Sitting<br>ECG: NR	Fragmented QRS in the 12 leads, ↑ (***p* < 0.001**)
Eyuboglu et al. (2019)	216	$\overline{\text{Age}}$ = 50.5 years<br>$\widetilde{\text{Age}}$ = 4.3 years<br><br>45.8% Female	Diabetes Mellitus (8%)<br>Smoke (18%)<br>Excluded HT and multiple cardiovascular diseases<br><br>No BMIs reported	NT: 61 (28.2%)<br>PHT: 155 (71.8%)<br>PIHT: 0<br>HT: 0	12-lead	NR	BP: 24-hour ambulatory measurements<br>ECG: NR	Fragmented QRS in the 12 leads, ↑ (***p* = 0.028**)
Eyuboglu et al. (2017)	548	$\overline{\text{Age}}_p$ = 49.0 years<br>$\widetilde{\text{Age}}_p$ = 5.0 years<br><br>42.0% Female	Coronary Artery Disease (15.7%)<br>Diabetes Mellitus (18.8%)<br>Smoke (20.1%)<br>Excluded multiple cardiovascular diseases<br><br>No BMIs reported	NT: 159 (29.0%)<br>PHT: 172 (31.4%)<br>PIHT: 0<br>HT: 217 (39.6%)	12-lead	NR	BP: NR<br>ECG: NR	Fragmented QRS in the 12 leads, ↑<br>HT vs. NT: ***p* < 0.001**<br>HT vs. PHT: NS<br>PHT vs. NT: NS
Akintunde et al. (2012)	210	$\overline{\text{Age}}_p$ = 56.0 years<br>$\widetilde{\text{Age}}_p$ = 12.9 years<br><br>52.9% Female	Newly diagnosed with HT<br>Smoke (5.7%)<br>Excluded multiple comorbidities	NT: 70 (33.3%)<br>PHT: 0<br>PHIT: 0<br>HT: 140 (66.7%)	12-lead<br><br>I<br>V1/2<br>V5/6	NR	BP: NR<br>ECG: NR	QRS Axis in the 12 leads (***p* < 0.001**)<br>Mean R wave Amplitude in Lead I, ↑ (***p* = 0.007**)<br>SV1/V2 + RV5/6, ↑ (***p* = 0.003**)

			$\overline{BMI}_p$ = 26.5 kg/m^2<br>$\widetilde{BMI}_p$ = 5.5 kg/m^2					
Sriratanaviri yakul (2010)	1,485	$\overline{Age}$ = 34.4 years<br>$\widetilde{Age}$ = 5.4 years<br><br>57.0% Female	No exclusions listed<br>Diabetes (1.7%)<br>Impaired fasting glucose (14.6%)<br>Hypercholesterolemia (56.4%)<br>Smoking (12.7%)<br>Obesity (19.4%)<br><br>$\overline{BMI}_p$ = 22.2 kg/m^2<br>$\widetilde{BMI}_p$ = 3.5 kg/m^2	NT: 1,316 (88.6%)<br>PHT: 0<br>PHIT: 0<br>HT: 169 (11.4%)	12-lead	NR	BP: Sitting<br>ECG: Supine	Q/QS wave Abnormality in the 12 leads, NS
Solanki et al. (2018)	214	$\overline{Age}_p$ = 39.9 years<br>$\widetilde{Age}_p$ = 7.4 years<br><br>43.5% Female	Diabetes Mellitus (20%)<br>Hyperlipidemia (5%)<br>Cardiac disease (4%)<br>Smoke (37%)<br>Alcohol Use (22%)<br>Multiple comorbidity exclusions<br><br>No BMIs reported	NT: 72 (33.6%)<br>PHT: 0<br>PHIT: 0<br>HT: 142 (66.4%)	12-lead<br><br>aVL<br>V3	NR	BP: NR<br>ECG: Supine	R wave Amplitude in Lead aVL, ↑ (***p* < 0.001**)<br>S wave Amplitude in Lead V3, ↑ (***p* < 0.001**)
Aeschbacher et al. (2016)	2,070	$\overline{Age}_p$ = 36.4 years<br>$\widetilde{Age}_p$ = NR<br><br>52.9% Female	Smoke (22.0%)<br>Excluded multiple comorbidities<br><br>$\overline{BMI}_p$ = 24.6 kg/m^2<br>$\widetilde{BMI}_p$ = 3.8 kg/m^2	NT: 1640 (79.2%)<br>PHT: 0<br>PHIT: 0<br>HT: 430 (20.8%)	I	NR	BP: Sitting & 24-hour ambulatory<br>ECG: NR	R wave Amplitude in Lead I, ↑ (***p* < 0.0001**)
					II			R wave Amplitude in Lead II, NS
					aVR			S wave Amplitude in Lead aVR, ↑ (***p* < 0.0001**)
					V1			S Wave Amplitude in Lead V1,↑ (***p* < 0.0001**)
					V2			S Wave Amplitude in Lead V2, ↑ (***p* < 0.0001**)
Anttila et al. (2010)	5,613	$\overline{Age}_p$ = 51.9 years<br>$\widetilde{Age}_p$ = 14.3 years	Smoke (21.6%)	NT: 3,938 (70.2%)<br>PHT: 0	12-lead<br><br>V2, V3	150 Hz	BP: Sitting<br>ECG: Supine	PRWP+ (Poor R wave Progression) in Leads V2,V3, ↑

		56.1% Female	Chronic Obstructive Pulmonary Disease (1.3%)<br>Diabetes Mellitus (5.3%)<br>Left or Right Ventricular Hypertrophy (14.6%)<br>Coronary Heart Disease (8.0%)<br>Myocardial Infarction (2.3%)<br>Anti-HT medication taken<br>Excluded cardiac conduction disorders<br><br>$\overline{BMI}_p$ = 27.0 kg/m^2<br>$\widetilde{BMI}_p$ = 4.6 kg/m^2	PHIT: 0<br>HT: 1,675 (29.8%)				Male: NS<br>Female: $\boldsymbol{p < 0.01}$

Table E. Summary of papers that evaluated T wave features. BMI = body mass index, NT = normotensive, PHT = prehypertensive, HT = hypertensive, PIHT = pregnancy-induced hypertension, NS = not significant, BP = blood pressure. $\bar{x}$ = mean of variable x reported by the article, where x can be BMI or age. $\tilde{x}$ = standard deviation reported by the article, where x can be BMI or age. $\bar{x}_p$= calculated pooled mean of variable x, where x can be BMI or age. $\tilde{x}_p$= calculated pooled standard deviation of variable x, where x can be BMI or age. NR = Not reported.

**Study**	**Sample Size**	**Age & Sex**	**# of Comorbidities**	**NT/PHT/HT**	**Leads**	**Sampling Frequency**	**Position During Collection**	**T wave Features, Significance with BP**
Hassing et al. (2019)	1,449	$\overline{Age}$ = 22.7 years<br>$\widetilde{Age}$ = 3.0 years<br><br>26.3% Female	After medical screening, only healthy included.<br><br>$\overline{BMI}_p$ for SBP: 22.7 kg/m^2<br>$\widetilde{BMI}_p$ for SBP: 2.5 kg/m^2	NT: 1,197 (82.6%)<br>PHT: 252 (17.4%)<br>(by systolic BP)<br>PIHT: 0<br>HT: 0	12-lead	NR	BP: Supine<br>ECG: Supine	Maximum Duration in the 12 leads, ↓ ($\boldsymbol{p < 0.001}$)<br>Minimum Duration in the 12 leads, NS<br>Dispersion in the 12 leads, NS

Aeschbacher et al. (2016)	2,070	$\overline{Age}_p$ = 36.4 years<br>$\widetilde{Age}_p$ = NR<br><br>52.9% Female	Smoke (22.0%)<br>Excluded multiple comorbidities<br><br>$\overline{BMI}_p$ = 24.6 kg/m^2<br>$\widetilde{BMI}_p$ = 3.8 kg/m^2	NT: 1640 (79.2%)<br>PHT: 0<br>PHIT: 0<br>HT: 430 (20.8%)	V1	NR	BP: Sitting & 24 hour ambulatory<br>ECG: NR	Amplitude in Lead V1, ↑ (***p* < 0.0001**)
					V2			Amplitude in Lead V2, ↑ (***p* < 0.0001**)
					V4			Amplitude in Lead V4, ↑ (***p* < 0.009**)
					V5			Amplitude in Lead V5 ↑ (***p* = 0.03**)
Mozos and Filimon (2013)	60	$\overline{Age}$ = 36 years<br>$\widetilde{Age}$ = 10 years<br><br>45.0% Female	Smoke (51.6%)<br>Excluded multiple comorbidities<br><br>$\overline{BMI}$ = 26 kg/m^2<br>$\widetilde{BMI}$ = 4.5 kg/m^2	NT: 53 (88.3%)<br>PHT: 0<br>PHIT: 0<br>HT: 7 (11.7%)	12-lead<br><br>V5	NR	BP: NR<br>ECG: NR	Amplitude in Lead V5, ↑ (***p* = 0.032**)<br>Maximum in Lead V5, NS
Pusuroglu et al. (2016)	184	$\overline{Age}_p$ = 52.2 years<br>$\widetilde{Age}_p$ = 10.1 years<br><br>59.2% Female	Diabetes Mellitus (17.9%)<br>Smoke (20.8%)<br>Excluded multiple comorbidities<br><br>$\overline{BMI}_p$ = 30.1 kg/m^2<br>$\widetilde{BMI}_p$ = 5.3 kg/m^2	NT: 43 (23.4%)<br>PHT: 0<br>PHIT: 0<br>HT: 141 (76.6%)	12-lead	NR	BP: NR<br>ECG: 24-hour holter	T wave Alternans (+) in the 12 leads,<br>(***p* = 0.006**)
Assanelli et al. (2013)	18,959	$\overline{Age}_p$ = 54.6 years<br>$\widetilde{Age}_p$ = 11.1 years<br><br>54.8% Female	Smoke (25.8%)<br>Diabetes Mellitus (8.1%)<br>Hyperlipidemia (30.1%)<br>Excluded multiple cardiovascular diseases<br><br>$\overline{BMI}_p$ = 28.1 kg/m^2<br>$\widetilde{BMI}_p$ = 4.8 kg/m^2	NT: 8,658 (45.7%)<br>PHT: 0<br>PHIT: 0<br>HT: 10,301 (54.3%)	12-lead	NR	BP: Supine<br>ECG: NR	T wave Axis Deviation (+) in the 12 leads,<br>(***p* = 0.033**)

Sriratanaviriyakul(2010)	1,485	$\overline{Age}$ = 34.4 years<br>$\widetilde{Age}$ = 5.4 years<br><br>57.0% Female	No exclusions listed<br>Diabetes (1.7%)<br>Impaired fasting glucose (14.6%)<br>Hypercholesterolemia (56.4%)<br>Smoking (12.7%)<br>Obesity (19.4%)<br><br>$\overline{BMI}_p$ = 22.2 kg/m^2<br>$\widetilde{BMI}_p$ = 3.5 kg/m^2	NT: 1,316 (88.6%)<br>PHT: 0<br>PHIT: 0<br>HT: 169 (11.4%)	12-lead	NR	BP: Sitting<br>ECG: Supine	T wave Inversion in the 12 leads, NS

Table F. Summary of papers that evaluated T wave peak to T wave end features. TpTe = T peak to T end, NT = normotensive, PHT = prehypertensive, HT = hypertensive, PIHT = pregnancy-induced hypertension, NS = not significant. $\bar{x}$ = mean of variable x reported by the article, where x can be BMI or age. $\tilde{x}$ = standard deviation reported by the article, where x can be BMI or age. $\bar{x}_{\text{p}}$= calculated pooled mean of variable x, where x can be BMI or age. $\tilde{x}_{\text{p}}$= calculated pooled standard deviation of variable x, where x can be BMI or age. NR = Not reported.

**Study**	**Sample Size**	**Age & Sex**	**# of Comorbidities**	**NT/PHT/HT**	**Leads**	**Sampling Frequency**	**Position During Collection**	**T wave Peak to T wave End Features, Significance with BP**
Hassing et al. (2019)	1,449	$\overline{\text{Age}}$ = 22.7 years<br>$\widetilde{\text{Age}}$ = 3.0 years<br><br>26.3% Female	After medical screening, only healthy included.<br><br>$\overline{\text{BMI}}_{\text{p}}$ for SBP: 22.7 kg/m^2<br>$\widetilde{\text{BMI}}_{\text{p}}$ for SBP: 2.5 kg/m^2	NT: 1,197 (82.6%)<br>PHT: 252 (17.4%)<br>(by systolic BP)<br>PIHT: 0<br>HT: 0	12-lead	NR	BP: Supine<br>ECG: Supine	TpTe in the 12 leads, NS
Gazi et al. (2016)	41	$\overline{\text{Age}}_{\text{p}}$ = 28.2 years<br>$\widetilde{\text{Age}}_{\text{p}}$ = 5.7 years<br><br>100.0% Female	No listed comorbidities<br>Multiple comorbidity exclusions<br>Pregnancy<br><br>No BMIs reported	NT: 24 (58.5%)<br>PHT: 0<br>PIHT: 17 (41.5%)<br>HT: 0	12-lead	NR	BP: NR<br>ECG: NR	TpTe in the 12 leads,↑ (***p* = 0.007**)<br>TpTe/QT in the 12 leads, NS<br>TpTe/QTc in the 12 leads, ↑ (***p* = 0.037**)
Kirbas et al. (2016)	96	$\overline{\text{Age}}_{\text{p}}$ = 27.6 years<br>$\widetilde{\text{Age}}_{\text{p}}$ = 5.1 years<br><br>100.0% Female	Pregnancy<br>Preeclampsia<br>Excluded multiple comorbidities<br><br>$\overline{\text{BMI}}_{\text{p}}$ = 29.3 kg/m^2<br>$\widetilde{\text{BMI}}_{\text{p}}$ = 3.5 kg/m^2	NT: 32 (33.3%)<br>PHT: 0<br>PIHT: 64 (66.6%)<br>HT: 0	12-lead<br><br>II, V2, V5	NR	BP: NR<br>ECG: NR	*(NT vs. mild preeclampsia;*<br>*NT vs. severe preeclampsia)*<br><br>TpTe in Leads II, V2, V5, ↑<br>**(*p* < 0.001, *p* < 0.001)**<br>TpTe/QTc in Leads II, V2, V5, ↑<br>**(*p* < 0.001, *p* < 0.001)**
Ferrucci et al. (2015)	50	$\overline{\text{Age}}$ = 42.8 years<br>$\widetilde{\text{Age}}$ = 9.1 years<br><br>Sex not described	No listed comorbidities<br>Multiple comorbidity exclusions<br>New HT diagnosis<br>No HT medications	NT: 18 (36.0%)<br>PHT: 0<br>PHIT: 0<br>HT: 32 (64.0%)	12-lead	NR	BP: Sitting<br>ECG: Supine	TpTe in the 12 leads,, ↑ (***p* < 0.001**)

			$\overline{\text{BMI}}$ = 26.1 kg/m^2<br>$\widetilde{\text{BMI}}$ = 4.0 kg/m^2					
Tanindi et al. (2015)	84	$\overline{\text{Age}}_p$ = 51.5 years<br>$\widetilde{\text{Age}}_p$ = 13.1 years<br><br>56.0% Female	No listed comorbidities<br>Multiple comorbidity exclusions<br><br>$\overline{\text{BMI}}_p$ = 26.3 kg/m^2<br>$\widetilde{\text{BMI}}_p$ = 2.9 kg/m^2	NT: 37 (44.0%)<br>PHT: 47 (56.0%)<br>PIHT: 0<br>HT: 0	12-lead	NR	BP: Sitting<br>ECG: Supine	TpTe in the 12 leads, ↑ (***p* < 0.001**)<br>TpTe/QT in the 12 leads, ↑ (***p* < 0.001**)
Mozos and Filimon (2013)	60	$\overline{\text{Age}}$ = 36 years<br>$\widetilde{\text{Age}}$ = 10 years<br><br>45.0% Female	Smoke (51.6%)<br>Excluded multiple comorbidities<br><br>$\overline{\text{BMI}}$ = 26 kg/m^2<br>$\widetilde{\text{BMI}}$ = 4.5 kg/m^2	NT: 53 (88.3%)<br>PHT: 0<br>PHIT: 0<br>HT: 7 (11.7%)	12-lead<br><br>V5	NR	BP: NR<br>ECG: NR	TpTe Maximum in Lead V5, ↑<br>(***p* = 0.001**)
Zhao et al. (2010)	121<br><br>(83 without LVH)	$\overline{\text{Age}}_p$ = 57.3 years<br>$\widetilde{\text{Age}}_p$ = 12.0 years<br><br>44.6% Female	No listed comorbidities<br>Multiple comorbidity exclusions<br><br>No BMIs reported	NT: 42 (50.6%)<br>PHT: 0<br>PHIT: 0<br>HT: 41 (49.4%)	12-lead<br><br>V4-V6	NR	BP: NR<br>ECG: NR	TpTe(c) in Leads V4 – V6, NS<br>TpTe(c)/QTc in Leads V4 – V6, NS

Table G. Summary of papers that evaluated other ECG morphologies. BMI = body mass index, NT = normotensive, PHT = prehypertensive, HT = hypertensive, PIHT = pregnancy-induced hypertension, NS = not significant, BP = blood pressure. $\bar{x}$ = mean of variable x reported by the article, where x can be BMI or age. $\tilde{x}$ = standard deviation reported by the article, where x can be BMI or age. $\bar{x}_p$ = calculated pooled mean of variable x, where x can be BMI or age. $\tilde{x}_p$ = calculated pooled standard deviation of variable x, where x can be BMI or age. NR = Not reported.

**Study**	**Sample Size**	**Age & Sex**	**# of Comorbidities**	**NT/PHT/HT**	**Leads**	**Sampling Frequency**	**Position During Collection**	**Other ECG wave Features, Significance with BP**
Hassing et al. (2019)	1,449	$\overline{\text{Age}}$ = 22.7 years<br>$\widetilde{\text{Age}}$ = 3.0 years	After medical screening, only healthy included.	NT: 1,197 (82.6%)	12-lead<br><br>V6	NR	BP: Supine<br>ECG: Supine	J point T peak Duration (corrected for HR) in the 12 leads, ↓<br>(***p* < 0.001**)

		26.3% Female	$\overline{BMI}_p$ for SBP: 22.7 kg/m^2<br>$\widetilde{BMI}_p$ for SBP: 2.5 kg/m^2	PHT: 252 (17.4%)<br>(by systolic BP)<br>PIHT: 0<br>HT: 0				VAT in Lead V6, ↑ (***p* < 0.001**)
Ferrucci et al. (2015)	50	$\overline{Age}$ = 42.8 years<br>$\widetilde{Age}$ = 9.1 years<br><br>Sex not described	No listed comorbidities<br>Multiple comorbidity exclusions<br>New HT diagnosis<br>No HT medications<br><br>$\overline{BMI}$ = 26.1 kg/m^2<br>$\widetilde{BMI}$ = 4.0 kg/m^2	NT: 18 (36.0%)<br>PHT: 0<br>PHIT: 0<br>HT: 32 (64.0%)	12-lead<br><br>V6	NR	BP: Sitting<br>ECG: Supine	VAT in Lead V6, NS
Anigbogu et al. (2012)	78	No mean age reported<br><br>Sex not described	Excluded any disease conditions aside from hypertension.<br>Excluded significant alcohol use, smoking, and medications which affected cardiovascular function.<br><br>No BMIs reported	NT: 39 (50.0%)<br>PHT: 0<br>PHIT: 0<br>HT: 39 (50.0%)	12-lead	NR	BP: Sitting<br>ECG: NR	ST Duration in the 12 leads, NS
Queen et al. (2012)	1,280	$\overline{Age}$ = 43 years<br>$\widetilde{Age}$ = NR<br><br>67.6% Female	Metabolic syndrome (43.4%)<br>Diabetes Mellitus (28.8%)<br>Smoke (27.5%)<br><br>$\overline{BMI}$ = 30 kg/m^2<br>$\widetilde{BMI}$ = NR	NT: 981 (76.6%)<br>PHT: 0<br>PHIT: 0<br>HT: 299 (23.4%)	12-lead	NR	BP: NR<br>ECG: Supine	Ischemic ECG Abnormalities in the 12 leads, (***p* < 0.001**)
Sriratanaviriyakul (2010)	1,485	$\overline{Age}$ = 34.4 years<br>$\widetilde{Age}$ = 5.4 years<br><br>57.0% Female	No exclusions listed<br>Diabetes (1.7%)<br>Impaired fasting glucose (14.6%)<br>Hypercholesterolemia (56.4%)<br>Smoking (12.7%)<br>Obesity (19.4%)	NT: 1,316 (88.6%)<br>PHT: 0<br>PHIT: 0<br>HT: 169 (11.4%)	12-lead	NR	BP: Sitting<br>ECG: Supine	ST Depression in the 12 leads, NS<br><br>AV Conduction Defect in the 12 leads:<br>Third Degree Block, NS<br>Second Degree Block, NS<br>First Degree Block, NS

			$\overline{\mathrm{BMI}}_p$ = 22.2 kg/m^2<br>$\widetilde{\mathrm{BMI}}_p$ = 3.5 kg/m^2					Ventricular Conduction Defect in the 12 leads:<br>Left Bundle Branch Block, NS<br>Right Bundle Branch Block, (***p* = 0.04**)

REFERENCES

1 Gazi, E. *et al.* Does pregnancy-induced hypertension affect the electrophysiology of the heart? *J. Obstet. Gynaecol.* **36**, 183–186, doi:https://doi-org.ezproxy.library.ubc.ca/10.3109/01443615.2015.1036401 (2016).

2 Kirbas, A. *et al.* Novel indexes of arrhythmogenesis in preeclampsia: QT dispersion, Tp-e interval, and Tp-e/QT ratio. *Pregnancy Hypertens.* **6**, 38–41, doi:https://doi-org.ezproxy.library.ubc.ca/10.1016/j.preghy.2016.01.002 (2016).

3 Tanindi, A., Alhan, A. & Tore, H. F. Tp-e/QT ratio and QT dispersion with respect to blood pressure dipping pattern in prehypertension. *Blood Press. Monit.* **20**, 69–73, doi:https://doi-org.ezproxy.library.ubc.ca/10.1097/mbp.0000000000000090 (2015).

4 Chávez, E. *et al.* P-wave dispersion: a possible warning sign of hypertension in children. *MEDICC Rev.* **16**, 31–36 (2014).

5 Kirbas, O. *et al.* P-wave duration changes and dispersion in preeclampsia. *Eur. J. Obstet. Gynecol. Reprod. Biol.* **183**, 141–145, doi:https://doi-org.ezproxy.library.ubc.ca/10.1016/j.ejogrb.2014.10.042 (2014).

6 Chávez, E., González, E. F., Llanes Mdel, C., Llanes, M. G. & García, Y. Dependence of P-wave dispersion on mean arterial pressure as an independent hemodynamic variable in school children. *Colomb. Med. (Cali)* **44**, 178–183 (2013).

7 Chávez, E. *et al.* Relationship between P wave dispersion, left ventricular mass index and blood pressure. *Arch. Argent. Pediatr.* **111**, 206–212, doi:https://doi-org.ezproxy.library.ubc.ca/10.5546/aap.2013.206 (2013).

8 Anigbogu, C. N., Isichei, C. V. & Ajuluchukwu, J. N. Blood pressure, heart rate, cardiovascular reflexes and electrocardiographic changes in some hypertensive Nigerians. *Niger. J. Physiol. Sci.* **27**, 23–27 (2012).

9 Yildirim, N. *et al.* Atrial electromechanical coupling interval and P-wave dispersion in patients with white coat hypertension. *Clin. Exp. Hypertens.* **34**, 350–356, doi:https://doi-org.ezproxy.library.ubc.ca/10.3109/10641963.2011.649933 (2012).

10 Zhao, Z., Yuan, Z., Ji, Y., Wu, Y. & Qi, Y. Left ventricular hypertrophy amplifies the QT, and Tp-e intervals and the Tp-e/ QT ratio of left chest ECG. *J. Biomed. Res.* **24**, 69–72, doi:https://doi-org.ezproxy.library.ubc.ca/10.1016/s1674-8301(10)60011-5 (2010).

11 Chobanian, A. V. *et al.* The Seventh Report of the Joint National Committee on prevention, detection, evaluation, and treatment of high blood pressure: the JNC 7 report. *JAMA* **289**, 2560–2572, doi:https://doi-org.ezproxy.library.ubc.ca/10.1001/jama.289.19.2560 (2003).

12 Hassing, G. J. *et al.* Blood pressure-related electrocardiographic findings in healthy young individuals. *Blood Press.*, 113–122, doi:https://doi-org.ezproxy.library.ubc.ca/10.1080/08037051.2019.1673149 (2020).

13 Bekar, L. *et al.* Presence of fragmented QRS may be associated with complex ventricular arrhythmias in patients with essential hypertension. *J. Electrocardiol.* **55**, 20–25, doi:https://doi-org.ezproxy.library.ubc.ca/10.1016/j.jelectrocard.2019.04.009 (2019).

14 Dzikowicz, D. J. & Carey, M. G. Obesity and hypertension contribute to prolong QRS complex duration among middle-aged adults. *Ann. Noninvasive Electrocardiol.* **24**, e12665, doi:https://doi-org.ezproxy.library.ubc.ca/10.1111/anec.12665 (2019).

15 Whelton, P. K. *et al.* 2017 ACC/AHA/AAPA/ABC/ACPM/AGS/APhA/ASH/ASPC/NMA/PCNA guideline for the prevention, detection, evaluation, and management of high blood pressure in adults: executive summary: a report of the American College of Cardiology/American Heart Association task force on clinical practice guidelines. *Hypertension* **71**, 1269–1324, doi:https://doi-org.ezproxy.library.ubc.ca/10.1161/HYP.0000000000000066 (2018).

16 Eyuboglu, M. & Akdeniz, B. Association between non-dipping and fragmented QRS complexes in prehypertensive patients. *Arq. Bras. Cardiol.* **112**, 59–64, doi:https://doi-org.ezproxy.library.ubc.ca/10.5935/abc.20180242 (2019).

17 Sun, G., Zhou, Y., Ye, N., Wu, S. & Sun, Y. Independent associations of blood pressure and body mass index with interatrial block: a cross-sectional study in general Chinese population. *BMJ Open* **9**, e029463, doi:https://doi-org.ezproxy.library.ubc.ca/10.1136/bmjopen-2019-029463 (2019).

18 Sun, G. Z., Zhou, Y., Ye, N., Wu, S. J. & Sun, Y. X. Independent influence of blood pressure on QTc interval: results from a general Chinese population. *Biomed. Res. Int.* **2019**, 1656123, doi:https://doi-org.ezproxy.library.ubc.ca/10.1155/2019/1656123 (2019).

19 Solanki, J. D. *et al.* Early screening of hypertension and cardiac dysautonomia in each hypertensive is needed-inference from a study of QTc interval in Gujarat, India. *Int. J. Prev. Med.* **9**, 62, doi:https://doi-org.ezproxy.library.ubc.ca/10.4103/ijpvm.ijpvm_423_15 (2018).

20 Tosun, V. *et al.* Evaluation of atrial electromechanical functions in dipper and nondipper hypertension patients using left atrial strain P-wave dispersion and P terminal force. *Echocardiography* **35**, 1318–1325, doi:https://doi-org.ezproxy.library.ubc.ca/10.1111/echo.14041 (2018).

21 Eyuboglu, M. *et al.* Usefulness of fragmented QRS in hypertensive patients in the absence of left ventricular hypertrophy. *J. Clin. Hypertens. (Greenwich)* **19**, 861–865, doi:https://doi-org.ezproxy.library.ubc.ca/10.1111/jch.13051 (2017).

22 Aeschbacher, S. *et al.* Relationships of electrocardiographic parameters with ambulatory hypertension in young and healthy adults. *Int. J. Cardiol.* **202**, 300-304, doi:https://doi-org.ezproxy.library.ubc.ca/10.1016/j.ijcard.2015.09.013 (2016).

23 Avci, B. K., Gulmez, O., Donmez, G. & Pehlivanoglu, S. Early changes in atrial electromechanical coupling in patients with hypertension: assessment by tissue doppler imaging. *Chin. Med. J. (Engl)* **129**, 1311–1315, doi:https://doi-org.ezproxy.library.ubc.ca/10.4103/0366-6999.182846 (2016).

24 Williams, B. *et al.* 2018 ESC/ESH Guidelines for the management of arterial hypertension: The Task Force for the management of arterial hypertension of the European Society of Cardiology and the European Society of Hypertension: The Task Force for the management of arterial hypertension of the European Society of Cardiology and the European Society of Hypertension. *J. Hypertens.* **36**, 1953–2041, doi:https://doi-org.ezproxy.library.ubc.ca/10.1097/hjh.0000000000001940 (2018).

25 Pusuroglu, H. *et al.* Assessment of relationship between galectin-3 and ambulatory ECG-based microvolt T-wave alternans in sustained systolic-diastolic hypertension patients. *Blood Press. Monit.* **21**, 265-270, doi:https://doi-org.ezproxy.library.ubc.ca/10.1097/mbp.0000000000000197 (2016).

26 Vaidean, G. D., Manczuk, M. & Magnani, J. W. Atrial electrocardiography in obesity and hypertension: clinical insights from the Polish-Norwegian study (PONS). *Obesity (Silver Spring)* **24**, 2608–2614, doi:https://doi-org.ezproxy.library.ubc.ca/10.1002/oby.21678 (2016).

27 Ferrucci, A. *et al.* A novel electrocardiographic T-wave measurement (Tp-Te interval) as a predictor of heart abnormalities in hypertension: a new opportunity for first-line electrocardiographic evaluation. *J. Clin. Hypertens. (Greenwich)* **17**, 441–449, doi:https://doi-org.ezproxy.library.ubc.ca/10.1111/jch.12522 (2015).

28 Mancia, G. *et al.* 2013 ESH/ESC guidelines for the management of arterial hypertension: the Task Force for the Management of Arterial Hypertension of the European Society of Hypertension (ESH) and of the European Society of Cardiology (ESC). *Eur. Heart. J.* **34**, 2159–2219, doi:https://doi-org.ezproxy.library.ubc.ca/10.1097/01.hjh.0000431740.32696.cc (2013).

29 Ale, O. K., Ajuluchukwu, J. N., Oke, D. A. & Mbakwem, A. C. QT dispersion in hypertensive Nigerians with and without left ventricular hypertrophy. *West Afr. J. Med.* **32**, 57–61 (2013).

30 Assanelli, D. *et al.* T-wave axis deviation and left ventricular hypertrophy interaction in diabetes and hypertension. *J. Electrocardiol.* **46**, 487–491, doi:https://doi-org.ezproxy.library.ubc.ca/10.1016/j.jelectrocard.2013.08.002 (2013).

31 Mozos, I. & Filimon, L. QT and Tpeak-Tend intervals in shift workers. *J. Electrocardiol.* **46**, 60–65, doi:https://doi-org.ezproxy.library.ubc.ca/10.1016/j.jelectrocard.2012.10.014 (2013).

32 Mozos, I., Costea, C., Serban, C. & Susan, L. Factors associated with a prolonged QT interval in liver cirrhosis patients. *J. Electrocardiol.* **44**, 105–108, doi:https://doi-org.ezproxy.library.ubc.ca/10.1016/j.jelectrocard.2010.10.034 (2011).

33 Akintunde, A. A., Oyedeji, A. T., Familoni, O. B., Ayodele, O. E. & Opadijo, O. G. QT interval prolongation and dispersion: epidemiology and clinical correlates in subjects with newly diagnosed systemic hypertension in Nigeria. *J. Cardiovasc. Dis. Res.* **3**, 290–295, doi:https://doi-org.ezproxy.library.ubc.ca/10.4103/0975-3583.102705 (2012).

34 Magnani, J. W. *et al.* P wave indices, obesity, and the metabolic syndrome: the Atherosclerosis Risk in Communities study. *Obesity (Silver Spring)* **20**, 666–672, doi:https://doi-org.ezproxy.library.ubc.ca/10.1038/oby.2011.53 (2012).

35 Soliman, E. Z., Prineas, R. J., Case, L. D., Zhang, Z. M. & Goff, D. C., Jr. Ethnic distribution of ECG predictors of atrial fibrillation and its impact on understanding the ethnic distribution of ischemic stroke in the Atherosclerosis Risk in Communities (ARIC) study. *Stroke* **40**, 1204–1211, doi:https://doi-org.ezproxy.library.ubc.ca/10.1161/strokeaha.108.534735 (2009).

36 Queen, S. R. *et al.* Electrocardiographic abnormalities among Mexican Americans: corrections with diabetes, obesity, and the metabolic syndrome. *World J. Cardiovasc. Dis.* **2**, 50–56, doi:https://doi-org.ezproxy.library.ubc.ca/10.4236/wjcd.2012.22009 (2012).

37 Baumert, M. *et al.* Relation between QT interval variability and cardiac sympathetic activity in hypertension. *Am. J. Physiol. Heart Circ. Physiol.* **300**, H1412–H1417, doi:https://doi-org.ezproxy.library.ubc.ca/10.1152/ajpheart.01184.2010 (2011).

38 Emiroglu, M. Y. *et al.* Assessment of atrial conduction time in patients with essential hypertension. *J. Electrocardiol.* **44**, 251–256, doi:https://doi-org.ezproxy.library.ubc.ca/10.1016/j.jelectrocard.2010.09.012 (2011).

39 Ermis, N. *et al.* Comparison of atrial electromechanical coupling interval and P-wave dispersion in non-dipper versus dipper hypertensive subjects. *Blood Press.* **20**, 60–66, doi:https://doi-org.ezproxy.library.ubc.ca/10.3109/08037051.2010.532302 (2011).

40 Pshenichnikov, I. *et al.* Association between ventricular repolarization and main cardiovascular risk factors. *Scand. Cardiovasc. J.* **45**, 33–40, doi:https://doi-org.ezproxy.library.ubc.ca/10.3109/14017431.2010.532232 (2011).

41 Anttila, I. *et al.* Prevalence and prognostic value of poor R-wave progression in standard resting electrocardiogram in a general adult population. The Health 2000 survey. *Ann. Med.* **42**, 123–130, doi:https://doi-org.ezproxy.library.ubc.ca/10.3109/07853890903555334 (2010).

42 Sriratanaviriyakul, N., Kangkagate, C. & Krittayaphong, R. Prevalences and association of ECG findings and cardiovascular risk factor in Shinawatra employees. *J. Med. Assoc. Thai.* **93 Suppl 1**, S1–S10 (2010).
